# Chiral metal–macrocycle frameworks: supramolecular chirality induction and helicity inversion of the helical macrocyclic structures†
†Electronic supplementary information (ESI) available: Spectroscopic analysis in solution, preparation and characterization of the crystals and crystallographic data. CCDC 989061–989070. For ESI and crystallographic data in CIF or other electronic format see DOI: 10.1039/c5sc04570c


**DOI:** 10.1039/c5sc04570c

**Published:** 2016-01-11

**Authors:** Ryou Kubota, Shohei Tashiro, Mitsuhiko Shionoya

**Affiliations:** a Department of Chemistry , Graduate School of Science , The University of Tokyo , 7-3-1 Hongo , Bunkyo-ku , Tokyo 113-0033 , Japan . Email: shionoya@chem.s.u-tokyo.ac.jp

## Abstract

Porous molecular solids composed of discrete macrocycles/cages have great potential for catalysis, separation and sensing techniques. Dynamic structural transformation of the host building blocks, especially a helicity inversion responsive to chemical triggers, is central to upgrading the spatial functions. Here we have achieved the syntheses of homochiral porous molecular solids composed of helical metal macrocycles through supramolecular chirality induction to both enantiomorphic forms with the aid of two different enantiopure sugar-derived lactones in the crystallization process. Moreover, we found that the helicity of the macrocyclic skeletons can be inverted in the crystalline state only by changing the type of solvent. This finding would lead to dynamic control of space chirality in connection with optical resolution, chiral amplification and asymmetric reactions.

## Abstract 2

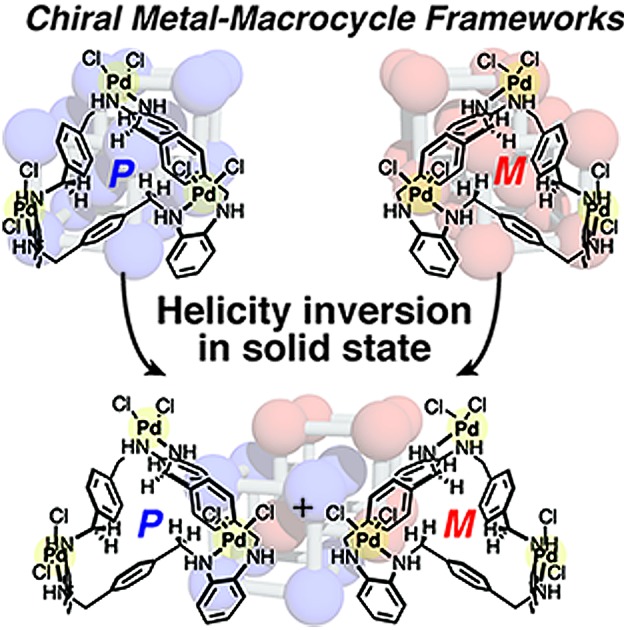
Homochiral metal–macrocycle frameworks have been synthesized through supramolecular chirality induction with the aid of enantiopure sugar-derived lactones.

## Introduction

Porous molecular solids (PMSs) composed of shape-persistent macrocycles/cages show promise for solid-state functions such as molecular storage, separation and catalysis.^1^,^2^ Through weak intermolecular interactions such as van der Waals attraction and hydrogen bonding, PMSs often show structural transformation in response to physical and chemical stimuli, enabling dynamic switching of porous functions.^3^ Such switching systems, however, are limited to on–off switching of the porosity, mainly because the structural transformation of PMSs is neither due to the conformational nor configurational change, but to the packing transformation of the components. As previously reported,^4^ the helicity of macrocyclic skeletons can be stably induced by metal complexation in solution. For example, the macrocyclic helicity can be inverted in response to chemical stimuli through spontaneous ligand exchange on the metal center.4b–f Thus, PMSs consisting of helical metal–macrocycles would have great potential not only for chiral spatial functions such as asymmetric catalysis and separation, but also for dynamic switching systems based on the helicity inversion of the macrocyclic scaffolds. However, such PMSs have not yet been explored so far, because, unless spontaneous crystallization happens, complexation of an *achiral* macrocycle and metal ions normally affords racemic crystals containing an equal amount of (*M*)- and (*P*)-forms of helical metal–macrocycles. Moreover, it is difficult to regulate asymmetric crystallization from an equilibrated mixture of helical macrocycles in a predictable manner, and it is even more challenging to control the chirality of porous crystals in the solid state.

**Fig. 1:**
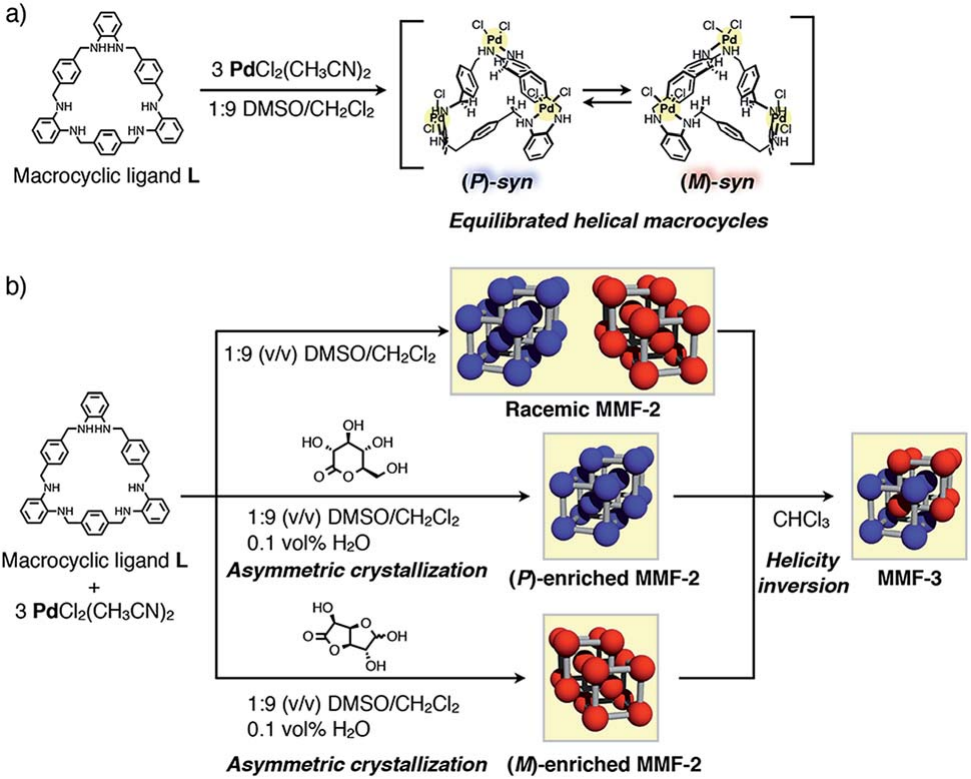
(a) Formation of (*P*)- and (*M*)-*syn* isomers of Pd_3_**L**Cl_6_ in equilibrium. (b) (*P*)- or (*M*)-enriched MMF-2 is formed by chiral induction of Pd_3_**L**Cl_6_ in the presence of an optically pure lactone. Without lactone, racemic MMF-2 is produced. MMF-2 is transformed into a racemic crystal, MMF-3, *via* the helicity inversion of Pd_3_**L**Cl_6_. Blue and red balls represent (*P*)- and (*M*)-*syn* isomers, respectively.

Herein, we report chiral metal–macrocycle frameworks (MMFs) formed through supramolecular chirality induction^5^ in the crystallization of an equilibrated racemic mixture of helical (*M*)- and (*P*)-forms of metallo-hosts prepared from an achiral macrocyclic ligand (Fig. 1). Recently, we have reported a metal–macrocycle framework (MMF-1) with five enantiomerically paired molecular binding sites, which has ability to site-selectively arrange guest molecules on the nanochannel surface.^6^ MMF-1 is composed of four stereoisomers of trinuclear Pd^II^ macrocyclic complexes, Pd_3_**L**Cl_6_, derived from conformation isomers (*syn* and *anti*) and helical enantiomers (*M* and *P*). As part of investigation of the solvent effect on the self-assembly processes, we found that only a pair of enantiomers, (*M*)- and (*P*)-*syn* isomers, were formed in a 1 : 9 (v/v) mixture of dimethylsulfoxide (DMSO)/CH_2_Cl_2_ to produce *racemic* MMF-2 (*rac*-MMF-2) as a racemic solid solution with a distinctly self-assembled porous structure (Fig. 1b). It is further worth noting that the chiral induction of MMF-2 was achieved by crystallization of Pd_3_**L**Cl_6_ in the presence of an optically-pure lactone. Moreover, we found that a crystal transformation takes place through helicity inversion of Pd_3_**L**Cl_6_ in a different solvent from (*M*)- or (*P*)-enriched MMF-2 to MMF-3 which has the same number of (*M*)- and (*P*)-*syn* isomers in a unit cell. This solvent-induced chirality transformation in the solid state would allow stimulus-triggered switching of spatial functions.

## Results and discussion

**Fig. 2:**
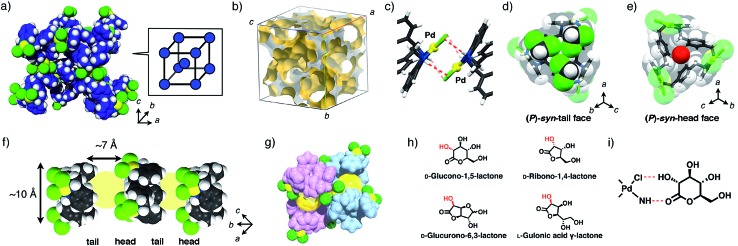
(a) A packing structure of (*P*)-MMF-2. The trinuclear Pd^II^ macrocyclic complexes, Pd_3_**L**Cl_6_, are located at the apex of a simple cubic lattice, and two simple cubic lattices interpenetrate with each other. Colors: C blue, N navy, Cl green, Pd yellow, H white. (b) A three-dimensional void structure of MMF-2.^11^ (c) Intermolecular Pd–Pd interactions between the neighboring Pd_3_**L**Cl_6_ complexes. (d) Three CH_2_Cl_2_ molecules and (e) one water molecule encapsulated in a (*P*)-*syn*-macrocyclic cavity at the tail and head pockets, respectively. (f) Cylindrically-shaped spaces (10 Å in diameter and 7 Å in length) put between the head and tail parts of the macrocyclic cavities. Colors: C gray, N navy, Cl green, Pd yellow, H white. (g) The arrangement of two cylindrically-shaped spaces. These spaces are interconnected through a small window. Colors: C, N and H pink or blue, Cl green, Pd yellow. (h) Chemical structures of optically-active lactones used in this study. (i) Possible interactions between Pd_3_**L**Cl_6_ and lactones.

In the crystallization screening by changing the type of solvents, we found that tetrahedron-shaped yellow single crystals, *rac*-MMF-2, composed of equal parts of (*M*)- and (*P*)-*syn* isomers were formed as a racemic solid solution in 47% yield upon complexation of ligand **L** and 3 equiv. of PdCl_2_(CH_3_CN)_2_ in a 1 : 9 (v/v) mixed solvent of DMSO/CH_2_Cl_2_.^7^*rac*-MMF-2 crystallized in a chiral space group *I*2_1_3 and each unit cell consisted of only one type of enantiomers, either (*M*)- or (*P*)-*syn* isomer, as determined by single-crystal X-ray analysis (Fig. 2a). The overall single-crystal, however, contained a pair of enantiomers (the Flack parameter^8^ = 0.50(5)), indicating that *rac*-MMF-2 is a racemic solid solution (Table 1).^9^ A unit cell of *rac*-MMF-2 contains two types of chemically-equivalent, but crystallographically-different *syn* isomers. These two *syn* isomers interact with each other through NH···Cl hydrogen bonding (N···Cl: 3.19 and 3.37 Å), CH···Pd nonclassical hydrogen bonding (C···Pd: 3.60 Å) and Pd–Pd interactions (3.20 Å apart) to form a three-dimensionally interconnected nano-sized pore (Fig. 2b and c). PLATON analysis^10^ indicated that the pore occupies 53% of the total volume of a unit cell (16 173 Å^3^/30 673 Å^3^). On the pore surface, two possible molecular binding pockets derived from a pair of the head and tail-sides of *syn* isomers face to each other to form a chiral cylindrically-shaped space (10 Å in diameter and 7 Å in length) that is put between a hydrophilic *syn*-head and a hydrophobic *syn*-tail faces (Fig. 2f). These chiral nano-spaces are connected through a small window formed between the interstitial voids of *syn* isomers (Fig. 2g). Moreover, a crystallographic analysis revealed that some of crystallization solvents, CH_2_Cl_2_ and H_2_O, are trapped on the rim and at the center of the amphiphilic binding pocket, respectively (Fig. 2d and e). This suggests a potential ability of *rac*-MMF-2 for guest sorting.

**Table 1:** Selected crystal parameters for asymmetric crystallization

	*R*_1_	w*R*_2_	Flack parameter
(*M*)-enriched MMF-2	0.0840	0.2381	0.06(7)
	0.1394	0.3554	0.07(10)
(*P*)-enriched MMF-2	0.0984	0.2726	0.07(9)
	0.0999	0.2627	0.04(9)
*rac*-MMF-2	0.0595	0.1548	0.50(5)

**Fig. 3:**
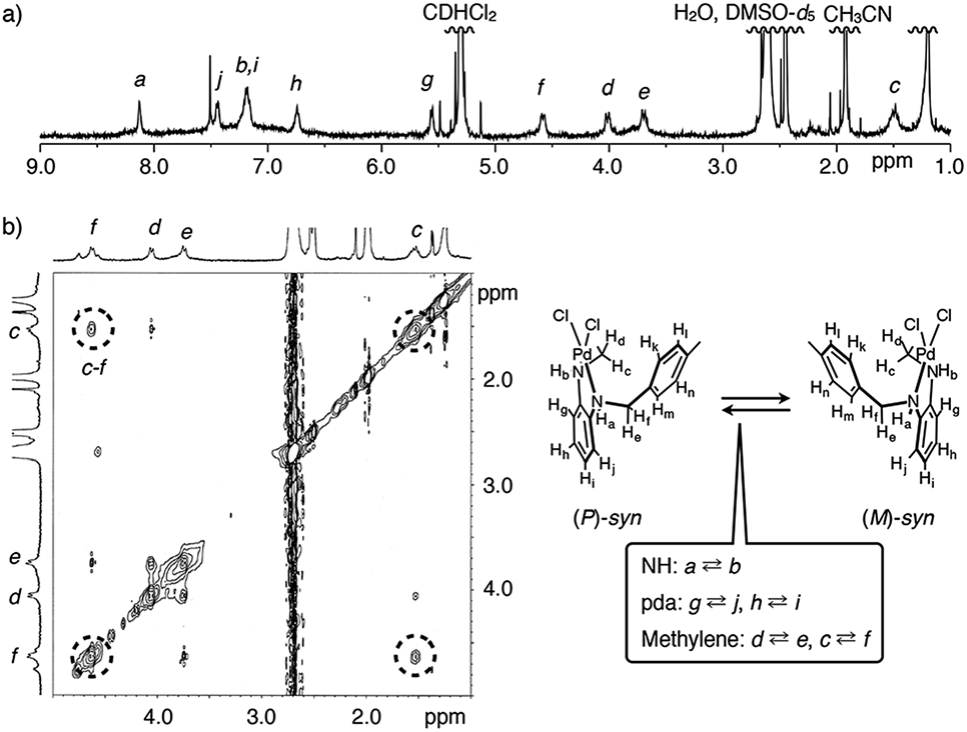
(a) ^1^H NMR spectrum of a mixture of ligand **L** and 3 equiv. of PdCl_2_(CH_3_CN)_2_ (500 MHz, 1 : 9 (v/v) DMSO-*d*_6_/CD_2_Cl_2_, 292 K, [**L**] = 1.1 mM). (b) ^1^H–^1^H EXSY spectrum of a mixture of ligand **L** and 3 equiv. of PdCl_2_(CH_3_CN)_2_ (500 MHz, 1 : 9 (v/v) DMSO-*d*_6_/CD_2_Cl_2_, 292 K, [**L**] = 1.1 mM, mixing time = 10 ms). pda: phenylenediamine.

To assess whether asymmetric crystallization of MMF-2 is possible or not, we examined the solution-phase behaviors of Pd_3_**L**Cl_6_ by ^1^H and 2D NMR spectroscopy. Upon addition of 3 equiv. of PdCl_2_(CH_3_CN)_2_ to ligand **L** in a 1 : 9 (v/v) mixed solvent of DMSO-*d*_6_/CD_2_Cl_2_, only (*P*/*M*)-*syn* isomers of Pd_3_**L**Cl_6_ were selectively formed (Fig. 1a), unlike in the case of complexation in CH_3_CN,6*a* as assigned by ^1^H–^1^H correlation spectroscopy (COSY) and rotating frame Overhauser enhancement spectroscopy (ROESY) (Fig. 3a and S3 and S4†). Notably, one of the methylene protons was shifted upfield by *ca.* 2.5 ppm, strongly indicating the helical structure formation in solution. We then estimated the conversion kinetics of the (*P*/*M*)-*syn* isomers by ^1^H–^1^H exchange spectroscopy (EXSY). Chemical exchange peaks were observed between methylene and phenylenediamine moieties, and thereby the kinetic constant of (*P*)–(*M*) helicity conversion of the (*P*/*M*)-*syn* isomers was determined to be 14 s^–1^ at 292 K (Fig. 3b and S5 and S6†). Thus, the (*P*)–(*M*) helical inversion was found to occur in solution at ambient temperature.

For the dynamic helicity inversion of Pd_3_**L**Cl_6_ in solution, we examined asymmetric crystallization of MMF-2 by adding an optically-pure lactone to a crystallization solution. As a result of adding d-glucurono-6,3-lactone (70 equiv.) or d-glucono-1,5-lactone (30 equiv.), extremely (*M*)- or (*P*)-enriched MMF-2 was obtained, respectively. The complexation of ligand **L** and 3 equiv. of PdCl_2_(CH_3_CN)_2_ in a 1 : 9 (v/v) mixed solvent of DMSO/CH_2_Cl_2_ at 25 °C in the presence of 0.1 vol% H_2_O and d-glucurono-6,3-lactone (70 equiv.) produced yellow tetrahedron-shaped block crystals of (*M*)-enriched MMF-2, Pd_3_**L**Cl_6_·(CH_2_Cl_2_)_6.9_, in 59% yield after one week. Single-crystal X-ray analysis of the crystal revealed that its unit cell structure was the same as that of the (*M*)-unit cell of *rac*-MMF-2. Notably, the Flack parameter was 0.06(7), which suggests that the asymmetry was amplified to an almost homochiral level (Table 1).^9^ To confirm the validity of asymmetric crystallization, we examined six more crystals which were randomly selected from several different batches. Consequently, all the crystals were extremely (*M*)-enriched MMF-2 with a Flack parameter of nearly zero (Table S5†), indicating that the result of asymmetric crystallization is reproducible.

When using d-glucono-1,5-lactone (30 equiv.) as a chiral induction reagent, yellow tetrahedron-shaped block crystals of exclusively (*P*)-enriched MMF-2, Pd_3_**L**Cl_6_·(CH_2_Cl_2_)_6.0_·(H_2_O), were produced in 36% yield. Its unit cell structure was the same as that of the (*P*)-unit cell of *rac*-MMF-2 and the Flack parameter was 0.07(9), which indicates that chirality induction was highly efficient (Table 1).^9^ We conducted single-crystal X-ray analysis using six crystals in total. Every structural analysis demonstrated that (*P*)-enriched crystals were generated reproducibly (Table S8†). Unfortunately, it was difficult to determine the optical purity of the bulk sample by solid-phase circular dichroism spectroscopy due to its very weak Cotton effect (Fig. S15†).

To quantify the amount of each chiral induction reagent in the solvent-accessible void and on the outer crystal surface, (*P*)- and (*M*)-enriched MMF-2 crystals prepared in the presence of an optically-pure lactone were isolated by filtration and then digested in DMSO-*d*_6_ without washing. Notably, as revealed by NMR spectroscopy, both as-synthesized crystals did not include any lactones. This result suggests that the lactones have almost no interactions with the pore and surface of the resulting crystals (Fig. S10 and S11†), but crystal growth experiments implies that the lactones play an essential role in the process (see the ESI†).

The effects of other optically-pure lactones on the chirality induction were also examined with d-ribono-1,4-lactone and l-gulonic acid γ-lactone. Crystallization in the presence of d-ribono-1,4-lactone (40 equiv.) or l-gulonic acid γ-lactone (18 equiv.) produced single-crystals of (*P*)- or (*M*)-enriched MMF-2, respectively. The Flack parameters were 0.25(9) and 0.17(8), respectively, suggesting a certain level of chiral induction. In the light of chemical structures of four lactones, the absolute configurations of the α-carbon of ester groups appear to have some correlations with the magnitude of chirality induction with MMF-2 crystals (Fig. 2h). Namely, d-glucurono-6,3-lactone and l-gulonic acid γ-lactone, whose α-carbons take an *S*-configuration, induce (*M*)-enriched MMF crystals, whereas d-glucono-1,5-lactone and d-ribono-1,4-lactone, whose α-carbons take an *R*-configuration, induce (*P*)-enriched MMF crystals. In contrast, natural sugars such as d-glucose and sucrose did not cause any significant chirality induction (Table S10†). This result suggests that the carbonyl and hydroxy groups on the α-carbon are key functionalities to achieve significant chirality induction largely due to hydrogen bonding between these functionalities and NH and Cl groups on the Pd centers as shown in Fig. 2i.

**Fig. 4:**
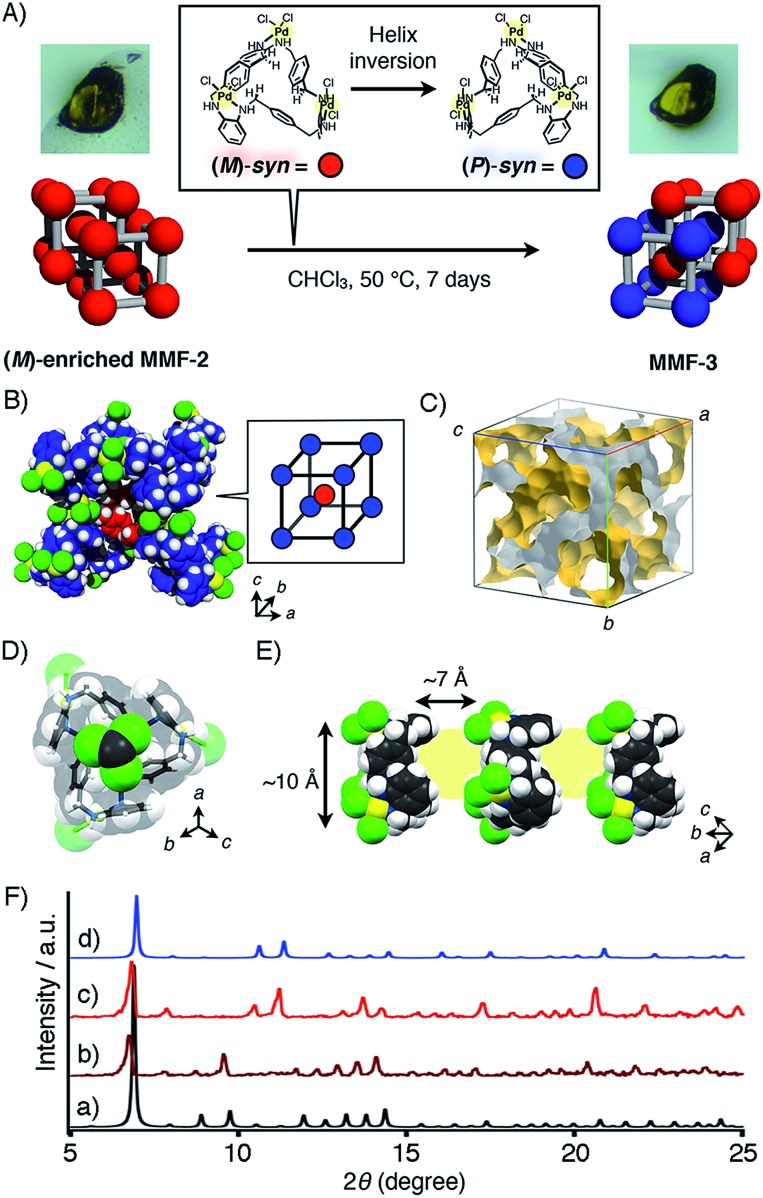
(A) Schematic illustration of the helical transformation from (*M*)-enriched MMF-2 to MMF-3 when (*M*)-enriched MMF-2 was soaked in CHCl_3_ at 50 °C. (B) A partial unit cell structure of MMF-3. Colors: N navy, Cl green, Pd yellow, H white. Carbon atoms of (*M*)- and (*P*)-*syn* enantiomers are depicted in red and blue, respectively. (C) The three-dimensional void structure of MMF-3.^11^ (D) An encapsulated CHCl_3_ molecule in the (*P*)-*syn*-tail pocket. (E) Cylindrical guest container space (*ca.* 10 nm in diameter and *ca.* 7 nm in length) put between the *syn*-head and -tail pockets. Colors: C gray, N navy, Cl green, Pd yellow, H white. (F) Powder XRD patterns of MMFs. Simulated diffraction patterns of (a) (*M*)-enriched MMF-2 and (d) MMF-3 calculated by single-crystal structures determined at –180 °C. Experimental diffraction patterns of (b) as-synthesized (*M*)-enriched MMF-2, (c) MMF-3 produced after soaking of (*M*)-enriched MMF-2 crystals in CHCl_3_ at 50 °C for 7 days.

As part of studies on the solvent effects on the chirality induction, we found that MMF-2 can be converted to a novel porous crystal, MMF-3. This conversion occurred through helicity inversion of the *syn* isomers only by soaking MMF-2 crystals in CHCl_3_ at 50 °C (Fig. 4A). For example, single-crystal X-ray analysis of (*M*)-enriched MMF-2 (Flack parameter: 0.04(12)) after soaking in CHCl_3_ at 50 °C for 7 days revealed that the structure was converted from (*M*)-enriched MMF-2 to MMF-3. The crystal system and unit cell size of MMF-3 were consistent with those of (*M*)-enriched MMF-2, but the space group was changed from *I*2_1_3 for (*M*)-enriched MMF-2 to *I*44̄33*d* for MMF-3 (Fig. 4B). A unit cell of MMF-3 is composed of both (*M*)- and (*P*)-*syn* isomers (*Z* = 16, eight molecules each). This means that almost half of the (*M*)-*syn* isomers of (*M*)-enriched MMF-2 crystals were transformed into the opposite (*P*)-*syn* isomers. Notably, the crystal morphology seems unchanged after the conversion, and therefore the crystal transformation may proceed in a single-crystal to single-crystal manner (Fig. 4A).

This observation suggests that MMF-3 is structurally more stable than MMF-2 in CHCl_3_ probably due to an increased number of NH···Cl hydrogen bonds and Pd–Pd interactions formed between Pd_3_**L**Cl_6_ units. In MMF-3, all the Pd–Cl moieties of the Pd centers interacted with each other in a head-to-tail manner through four NH···Cl hydrogen bonds (3.22 and 3.37 Å) and one Pd–Pd interaction (3.27 Å). In the case of MMF-2, on the other hand, only one type of the *syn* enantiomer interacted with each other in the same manner as MMF-3, while the other crystallographically-different isomer has a different association motif with two CH···Cl nonclassical hydrogen bonds and no Pd–Pd interactions (Fig. S16†). Thus, the transformation from MMF-2 to MMF-3 is well explained by such multipoint interactions due to a larger number of hydrogen bonds and Pd–Pd interactions in less polar CHCl_3_.

We then evaluated the crystal transformation of bulk MMF-2 crystals by powder XRD analysis. In the simulated powder XRD patterns of MMF-2 and MMF-3 drawn from their single-crystal structures, a major difference was found around 2*θ* = 10° (Fig. 4Fa and d). The powder XRD pattern of as-synthesized (*M*)-enriched MMF-2 was consistent with the simulated pattern, which demonstrates the phase purity of (*M*)-enriched MMF-2 (Fig. 4Fb). The powder XRD of (*M*)-enriched MMF-2 crystals heated in CHCl_3_ at 50 °C for a week revealed that the powder pattern of the resulting bulk powder was in good accordance with that of MMF-3, where the diffractions around 8 and 9° completely disappeared. This indicates that the crystal transformation from (*M*)-enriched MMF-2 to MMF-3 was fully converged (Fig. 4Fc). Furthermore, it was found that a racemic solid solution, *rac*-MMF-2, was also converted to MMF-3 under the same experimental condition (Fig. S19†).

## Conclusions

In summary, we have accomplished supramolecular chirality induction to both enantiomorphic forms in the crystallization of an equilibrated mixture of enantiomeric helical isomers of macrocycles, Pd_3_**L**Cl_6_, with the aid of two different enantiopure sugar-derived lactones. The chirality induction has reached an almost homochiral level. Moreover, the helicity of the macrocyclic skeleton was successfully inverted in the crystalline state only by changing the type of solvent. Thus, the dynamic chirality interconversion can be induced to both enantiomorphic forms in response to enantiopure additives and crystallization solvents in the crystalline state. The present protocol would be utilized not only for growing homochiral porous crystals but also for expanding dynamic chirality-based functions related to optical resolution and asymmetric reactions.

## Supplementary Material

Supplementary information

Crystal structure data
