# Supplementary material for: Chiral metal–macrocycle frameworks: supramolecular chirality induction and helicity inversion of the helical macrocyclic structures
Source: Chem Sci. 2016 Jan 11;7(3):2217–21. doi: 10.1039/c5sc04570c (PMC5975940; doi:10.1039/c5sc04570c)
Supplement: Supplementary file 1 [file SC-007-C5SC04570C-s001.pdf]

Electronic Supplementary Material (ESI) for Chemical Science.
This journal is © The Royal Society of Chemistry 2015

Electronic Supplementary Information

Chiral Metal-Macrocycle Frameworks: Supramolecular Chirality Induction and Helicity Inversion of the Helical Macrocyclic Structures

*Ryou Kubota, Shohei Tashiro and Mitsuhiko Shionoya**

Department of Chemistry, Graduate School of Science, The University of Tokyo,
7-3-1 Hongo, Bunkyo-ku, Tokyo 113-0033, Japan

correspondence to: shionoya@chem.s.u-tokyo.ac.jp

Table of contents

1. Materials and methods S2
2. Structural analyses of $Pd_3\mathbf{L}Cl_6$ in solution S3
3. Synthesis and characterization of *rac*-MMF-2 S13
4. Synthesis and characterization of (*M*)-enriched MMF-2 S16
5. Synthesis and characterization of (*P*)-enriched MMF-2 S21
6. Asymmetric crystallization of MMF-2 using other enantiopure compounds S25
7. Crystal growth experiments S29
8. Synthesis and characterization of MMF-3 S34
9. References for electronic supplementary information S38

1. Materials and Methods

Solvents and reagents were purchased from TCI Co., Ltd., Sigma-Aldrich Co., Kanto chemical Co., Inc. and WAKO Pure Chemical Industries Ltd. All chemicals were used without further purification. All ^{1}H NMR spectra were recorded on a Bruker AVANCEIII 500 and a Bruker DRX 500 (500 MHz) spectrometers. The residual solvents are used as the internal standard for ^{1}H NMR (DMSO-d_6: 2.50 ppm for DMSO-d_5, 1:9 (v/v) DMSO-d_6/CD_2Cl_2: 5.31 ppm for $CDHCl_2$). Infrared (IR) spectra were obtained with a JASCO FT/IR 4200 spectrometer using a ZnSe ATR method. UV-vis absorption spectra were recorded on a HITACHI U-3500 spectrophotometer. Thermogravimetric (TG) analyses were conducted with a Rigaku Thermo plus TG8120. Elemental analyses were performed on a YANAKO MT-6. Solution-phase and diffuse reflectance CD measurements were carried out with a JASCO J-820 spectropolarimeter. Powder X-ray analyses were conducted using a Rigaku Miniflex diffractometer with CuKα radiation. Microwave irradiation experiments were performed using an Anton Paar GmbH Monowave 300 single-mode microwave reactor. Single-crystal X-ray crystallographic analyses were performed using a Rigaku RAXIS-RAPID imaging plate diffractometer with MoKα radiation, and obtained data were calculated using the CrystalStructure crystallographic software package except for refinement, which was performed using SHELXL-97.[1] Some of solvent and guest molecules in all the crystal structures were

highly disordered. The volume of the solvent-accessible void was calculated with the program PLATON[2] using crystal structures omitting all solvent molecules. Several restrains (bond distances, angles and thermal parameters) were applied to solvent and guest molecules in all the crystal structures. All of the non-hydrogen atoms of Pd_3**L**Cl_6 and one $CHCl_3$ molecule in MMF-3 were refined anisotropically, while those of other solvent and guest molecules were refined isotropically. Hydrogen atoms were placed at the calculated positions and refined using a riding model. In the crystallographic analyses of MMF-2, we took account of two types of twinning by merohedry, twinning by inversion and rotation with twin laws (010, 100, 00–1), (0–10, –100, 00–1) or (–100, 00–1, 0–10), simultaneously. The X-ray structures were displayed using Mercury and CrystalExplorer.[3] The X-ray crystallographic coordinates for structures reported in this paper have been deposited at the Cambridge Crystallographic Data Centre under deposition numbers CCDC 989061 (*rac*-MMF-2), 989062 ((*P*)-enriched MMF-2), 989063 ((*M*)-enriched MMF-2), 989064 ((*P*)-enriched MMF-2 formed in the presence of D-ribono-1,4-lactone), 989065 ((*M*)-enriched MMF-2 formed in the presence of L-gulonic acid γ-lactone), 989066 (MMF-3), 989067 ((*M*)-enriched MMF-2 as a seed crystal for a crystal growth experiment), 989068 ((*M*)-enriched MMF-2 after crystal growth), 989069 (*rac*-MMF-2 as a seed crystal for a crystal growth experiment) and 989070 (*rac*-MMF-2 after crystal growth). These data can be obtained free of charge from the Cambridge Crystallographic Data Centre (http://www.ccdc.cam.ac.uk/data_request/cif).

2. Structural analyses of Pd_3LCl_6 in solution

*Preparation of Pd_3**L**Cl_6 in 1:9 (v/v) DMSO-d_6/CD_2Cl_2*

To a CD_2Cl_2 solution of ligand **L** (10.0 mM, 55 μL, 0.55 μmol) were added a DMSO-d_6 solution of $PdCl_2(CH_3CN)_2$ (50.0 mM, 31 μL, 1.55 μmol, 2.8 equiv.), DMSO-d_6 (22 μL), and CD_2Cl_2 (425 μL), and the mixture was allowed to stand for 5 min at room temperature ([**L**] = 1.03 mM). Its ^{1}H NMR spectrum showed a formation of Pd_3**L**Cl_6.

^{1}H NMR (500 MHz, 1:9 (v/v) DMSO-d_6/CD_2Cl_2, 293 K): δ 8.13 (br, 3H), 7.45-7.44 (d, J = 7.0 Hz 1H), 7.23-7.15 (m, 6H), 6.76-6.73 (dd, J = 7.0, 7.0 Hz 3H), 5.56 (d, J = 7.4 Hz, 3H), 4.59 (br, 3H), 4.01 (d, J = 12.5 Hz, 3H), 3.70 (d, J = 12.5 Hz, 3H), 1.48 (br, 3H).

^{1}H EXSY NMR measurements

^{1}H EXSY NMR measurements were conducted using a Bruker DRX500 (500 MHz) spectrometer at 292 K using the standard pulse sequence for phase-sensitive NOESY experiments programmed in XWinNMR Bruker software. A relaxation decay (D1) was set to 2 s, and various mixing times (τ_m: 5~400 ms) were chosen.

From EXSY measurements, if populations of both exchangeable species are equal, rate constants of exchange processes between two spectroscopically-distinct states (*k*) are derived according to the equation:[4]

$$k = \frac{1}{\tau_m} \ln \frac{r+1}{r-1}$$

$$r = \frac{I_{AA} + I_{BB}}{I_{AB} + I_{BA}}$$

where τ_m is mixing time of measurement, and I_{AA} and I_{BB} are the intensities of diagonal peaks, and I_{AB} and I_{BA} are those of cross peaks. The intensities of diagonal and cross peaks were calculated by using Topspin 3.0 after phase and baseline corrections in both dimensions. Using the calculated intensities, the rate constant (*k*) was estimated from the slope of the plots of ln[*r* + 1]/[*r* – 1] versus mixing time at short mixing time values.[5]

Titration experiments of D*-glucurono-6,3-lactone or* D*-glucono-1,5-lactone into $Pd_3\mathbf{L}Cl_6$ in a 1:9 (v/v) mixture of DMSO-d_6/CD_2Cl_2*

To a CD_2Cl_2 solution of ligand **L** (10 mM, 60 μL, 0.60 μmol) were added a DMSO-d_6 solution of $PdCl_2(CH_3CN)_2$ (50.7 mM, 38 μL, 1.93 μmol, 3.2 equiv.), DMSO-d_6 (20 μL), and CD_2Cl_2 (462 μL). To the resulting solution was added an appropriate amount of a DMSO-d_6 solution of D-glucurono-6,3-lactone (1.81 M) and CD_2Cl_2 to keep the solvent ratio (DMSO-d_6/CD_2Cl_2 = 1:9) constant.

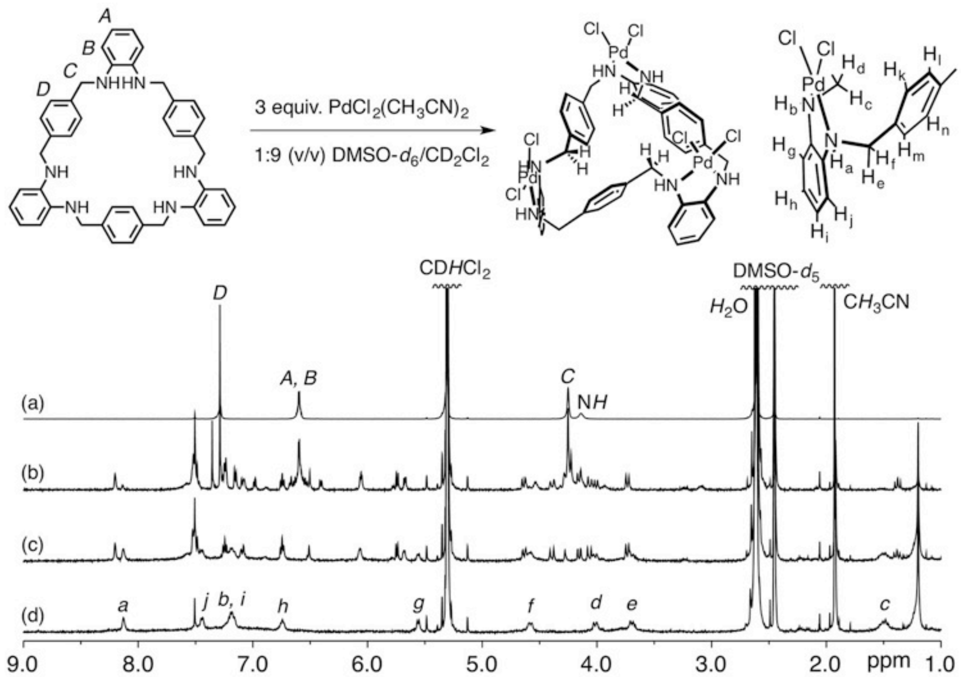

Figure S1. ^{1}H NMR spectra of ligand **L** with different amounts of $PdCl_2(CH_3CN)_2$ (500 MHz, 1:9 (v/v) DMSO-d_6/CD_2Cl_2, 293 K). (a) [**L**] = 1.21 mM; (b) [**L**] = 0.97 mM, $PdCl_2(CH_3CN)_2$ = 1.0 equiv.; (c) [**L**] = 0.81 mM, $PdCl_2(CH_3CN)_2$ = 2.0 equiv.; (d) [**L**] = 0.70 mM, $PdCl_2(CH_3CN)_2$ = 3.0 equiv. The intensity of spectrum (a) is decreased to one-tenth.

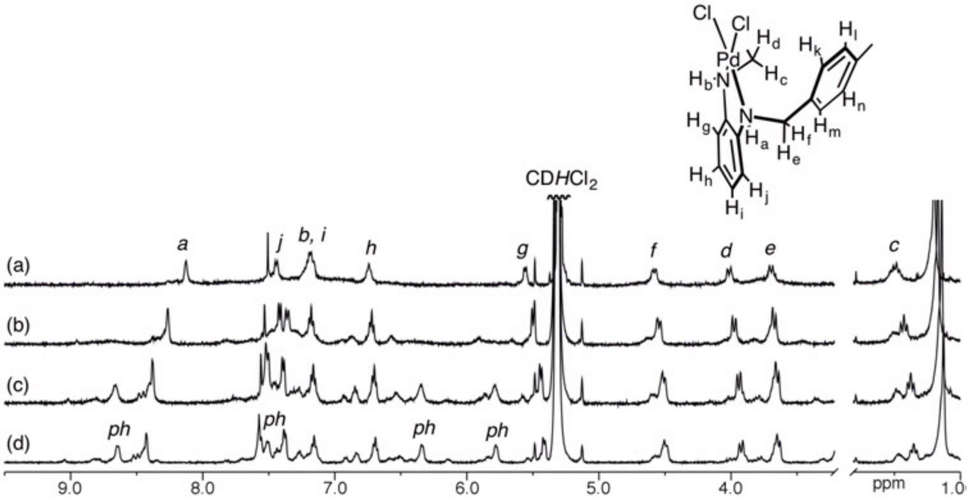

Figure S2. Variable temperature ^{1}H NMR spectra of a mixture of ligand **L** and 3 equiv. of $PdCl_2(CH_3CN)_2$ (500 MHz, 1:9 (v/v) DMSO-d_6/CD_2Cl_2, [**L**] = 0.70 mM). (a) 293; (b) 273; (c) 253; (d) 243 K. The signals derived from *p*-phenylene moieties were highly

broadened at 293 K due to the rotational motion, whereas the four *ph* signals newly observed at lower temperature can be assigned to signals of the phenylene moiety, *k*-*n*.

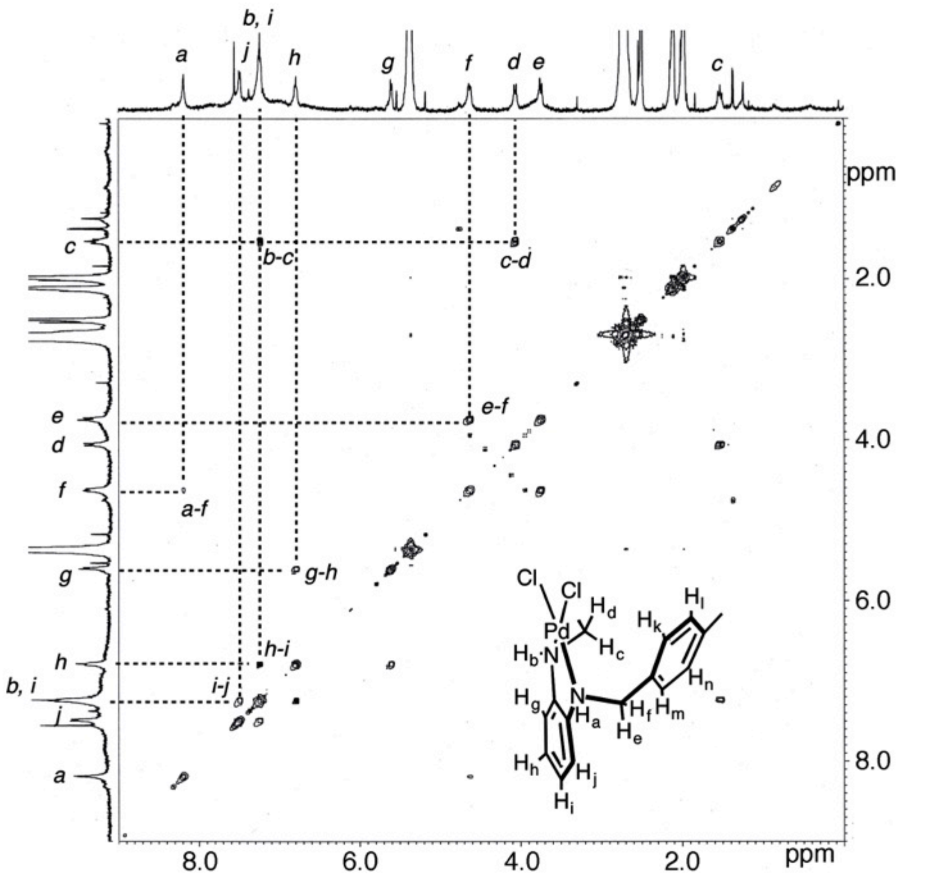

Figure S3. ^{1}H-^{1}H COSY spectrum of a mixture of ligand **L** and 3 equiv. of $PdCl_2(CH_3CN)_2$ (500 MHz, 1:9 (v/v) DMSO-d_6/CD_2Cl_2, 292 K, [**L**] = 1.1 mM).

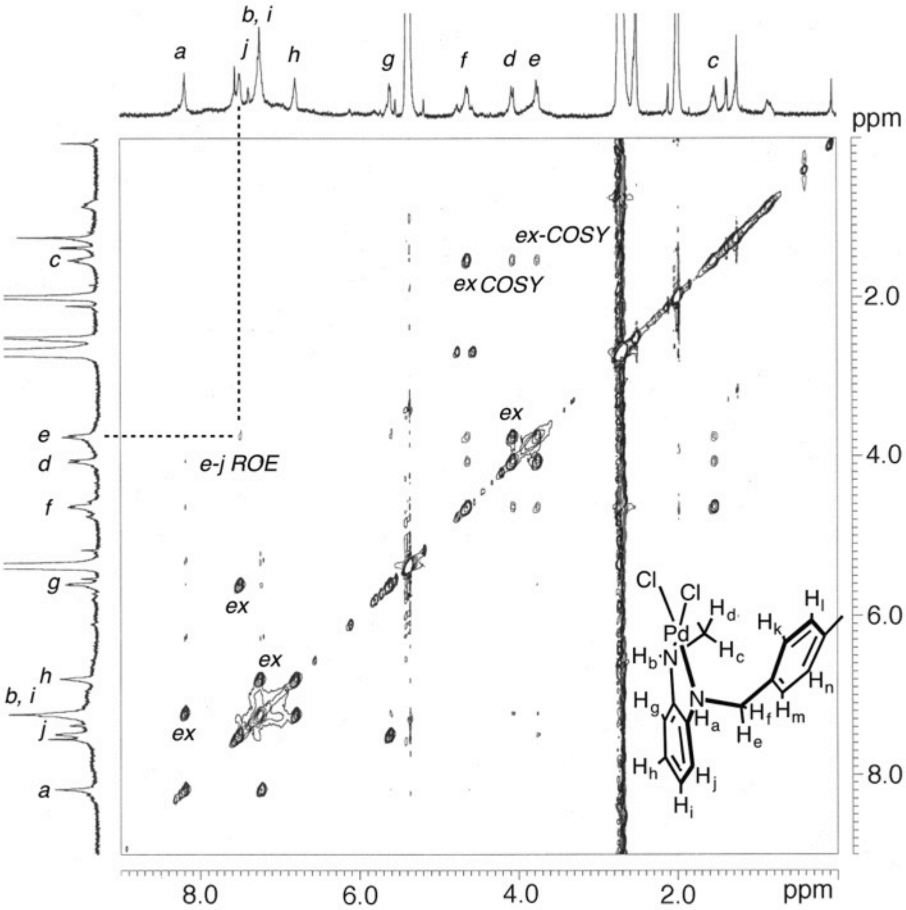

Figure S4. ^1H-^1H ROESY spectrum of a mixture of ligand **L** and 3 equiv. of $PdCl_2(CH_3CN)_2$ (500 MHz, 1:9 (v/v) DMSO-d_6/CD_2Cl_2, 292 K, [**L**] = 1.1 mM). *ex*: chemical exchange peaks.

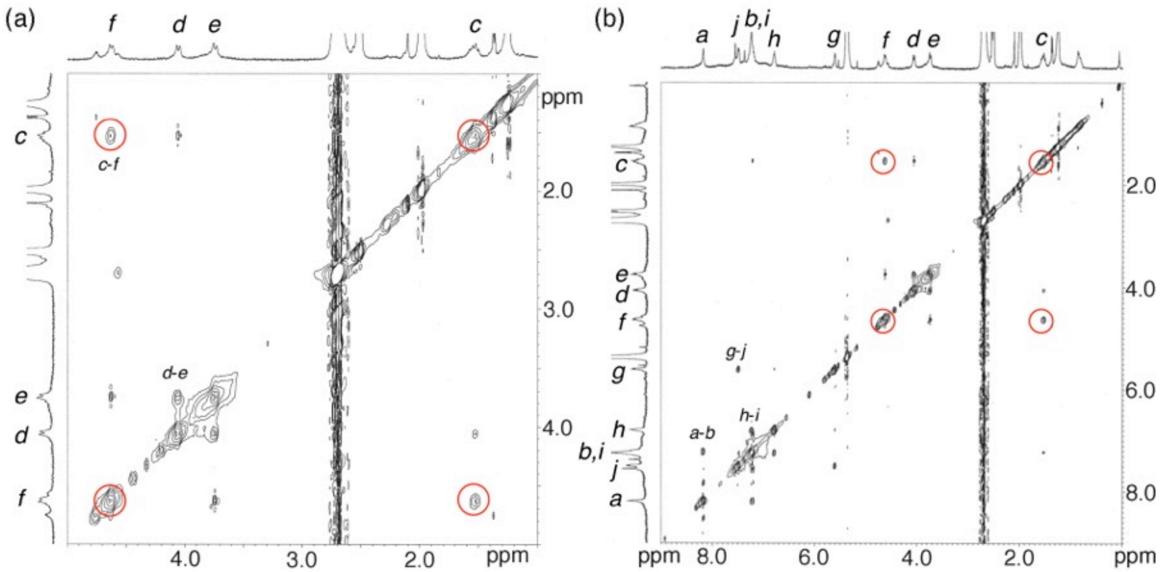

Figure S5. ^{1}H-^{1}H EXSY spectra of a mixture of ligand **L** and 3 equiv. of $PdCl_2(CH_3CN)_2$ (500 MHz, 1:9 (v/v) DMSO-d_6/CD_2Cl_2, 292 K, [**L**] = 1.1 mM, mixing time τ_m = 10 ms). (a) An enlarged spectrum around methylene signals, (b) the overall spectrum. Red circles represent peaks used for the calculation of an exchange rate constant.

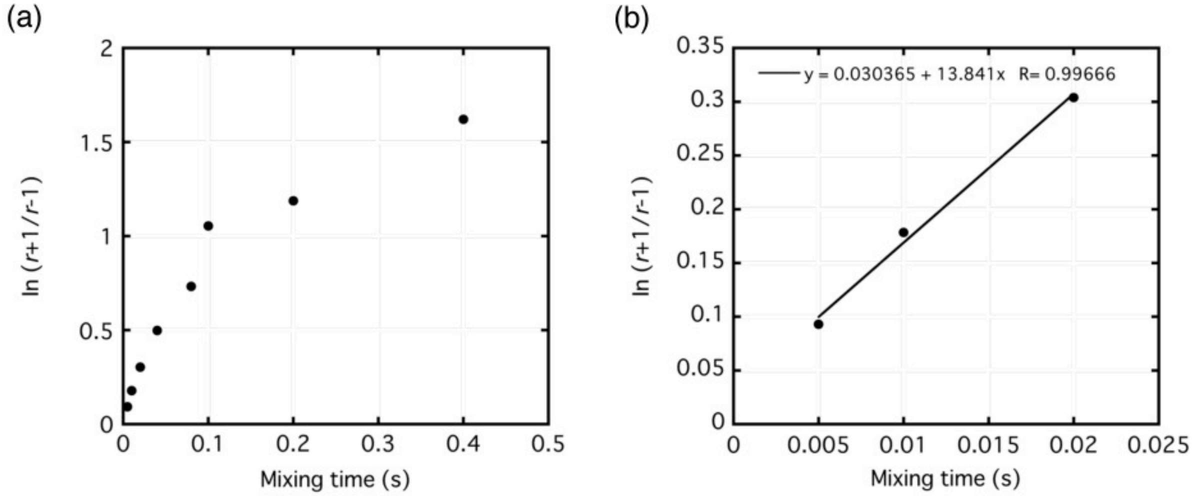

Figure S6. Plot of ln[(r + 1)/(r – 1)] *vs* mixing time τ_m for a mixture of ligand **L** and 3 equiv. of $PdCl_2(CH_3CN)_2$ from ^{1}H-^{1}H EXSY spectra (500 MHz, 1:9 (v/v) DMSO-d_6/CD_2Cl_2, 292 K) recorded with different mixing times.

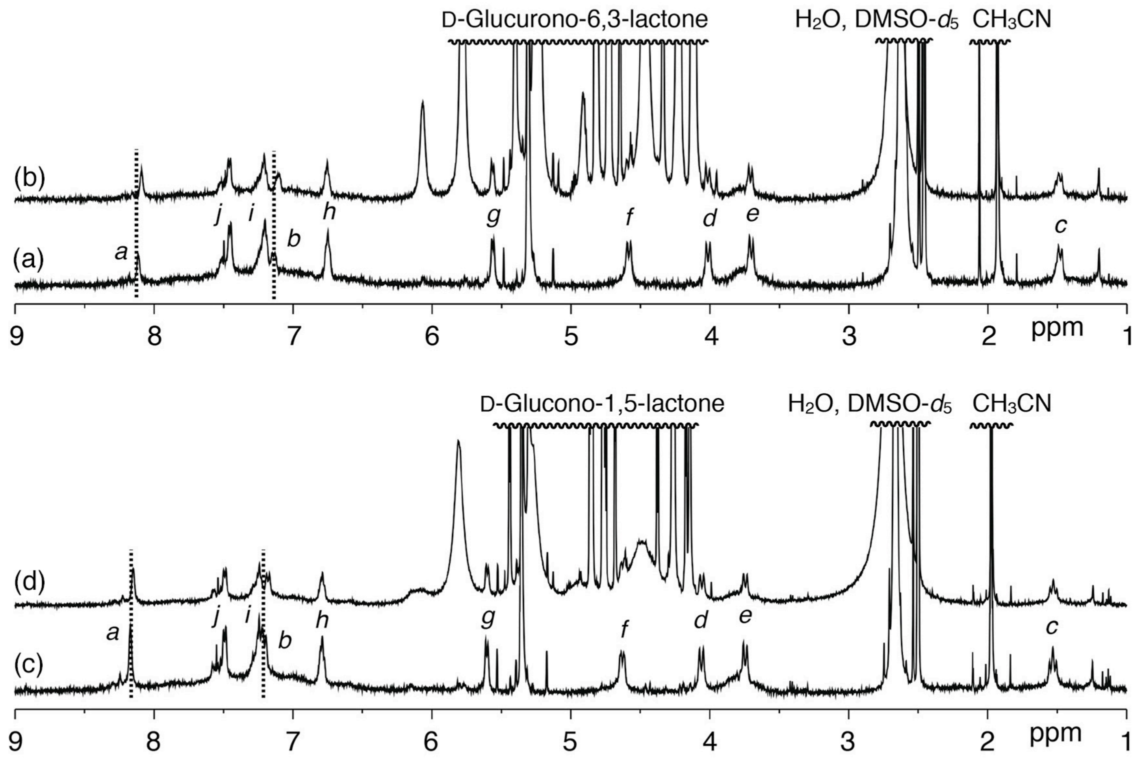

Figure S7. ^{1}H NMR spectra of a mixture of ligand **L** and 3 equiv. of $PdCl_2(CH_3CN)_2$ in the (a,c) absence or presence of (b) D-glucuruno-6,3-lactone (70 equiv.) or (d) D-glucono-1,5-lactone (30 equiv.) (500 MHz, 1:9 (v/v) DMSO-d_6/CD_2Cl_2, 292 K, [**L**] = 1.1 mM). No signal shifts due to diastereomeric aggregation were observed in the ^{1}H NMR spectra except a slight upfield shift of the amine proton signals. This result indicates that the interactions between the chirality induction reagents and the *syn*-Pd_3**L**Cl_6 are too weak to change the balance of the equilibrium.

*Solution-phase CD measurements of a mixture of Pd_3**L**Cl_6 and D-glucurono-6,3-lactone or D-glucono-1,5-lactone in 1:9 (v/v) DMSO/CH_2Cl_2*

A DMSO solution of Pd_3**L**Cl_6 was prepared by dissolving ligand **L** (1.15 mg, 1.82 μmol) into a DMSO solution of $PdCl_2(CH_3CN)_2$ (49.8 mM, 110 μL, 5.48 μmol, 3.01 equiv.). To this DMSO solution of Pd_3**L**Cl_6 (16.6 mM, 48 μL, 0.79 μmol) was added a DMSO solution of D-glucurono-6,3-lactone (1.81 M, 30.5 μL, 55.2 μmol, 70 equiv.), DMSO (20.5 μL) and CH_2Cl_2 (895 μL). The resulting solution was analyzed by solution-phase CD spectroscopy.

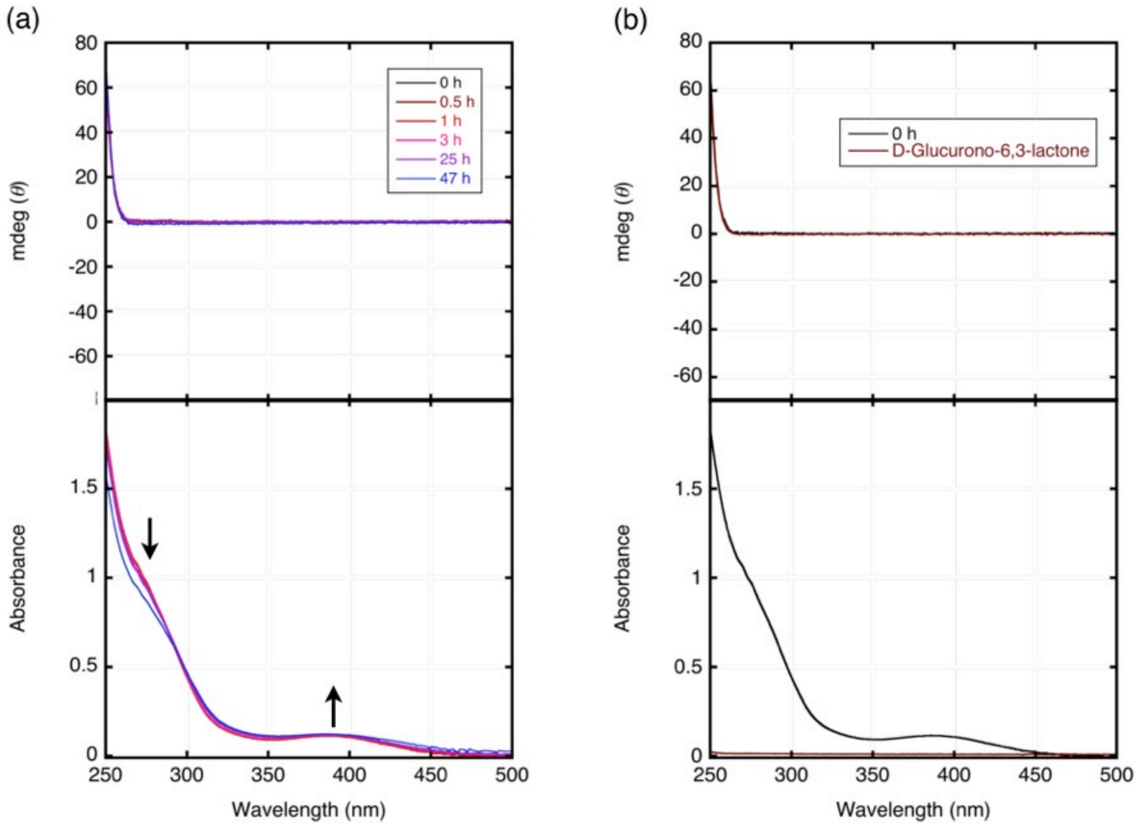

Figure S8. Solution-phase CD and UV-vis absorption spectra of a solution containing ligand **L**, 3 equiv. of $PdCl_2(CH_3CN)_2$ and 70 equiv. of D-glucurono-6,3-lactone ([**L**] = 0.8 mM, 25 °C, 1:9 (v/v) DMSO/CH_2Cl_2, *l* = 0.1 cm). (a) Time course measurements, (b) comparison between a solution of ligand **L**, 3 equiv. of $PdCl_2(CH_3CN)_2$ and 70 equiv. of D-glucurono-6,3-lactone and that of D-glucurono-6,3-lactone only ([D-glucurono-6,3-lactone] = 56 mM, 25 °C, 1:9 (v/v) DMSO/CH_2Cl_2, *l* = 0.1 cm). No chirality induction effects were confirmed by solution-phase CD spectroscopy. No Cotton effects were observed in the region from 270 nm to 450 nm due to the absorption of Pd_3**L**Cl_6 in the presence of D-glucurono-6,3-lactone or D-glucono-1,5-lactone.

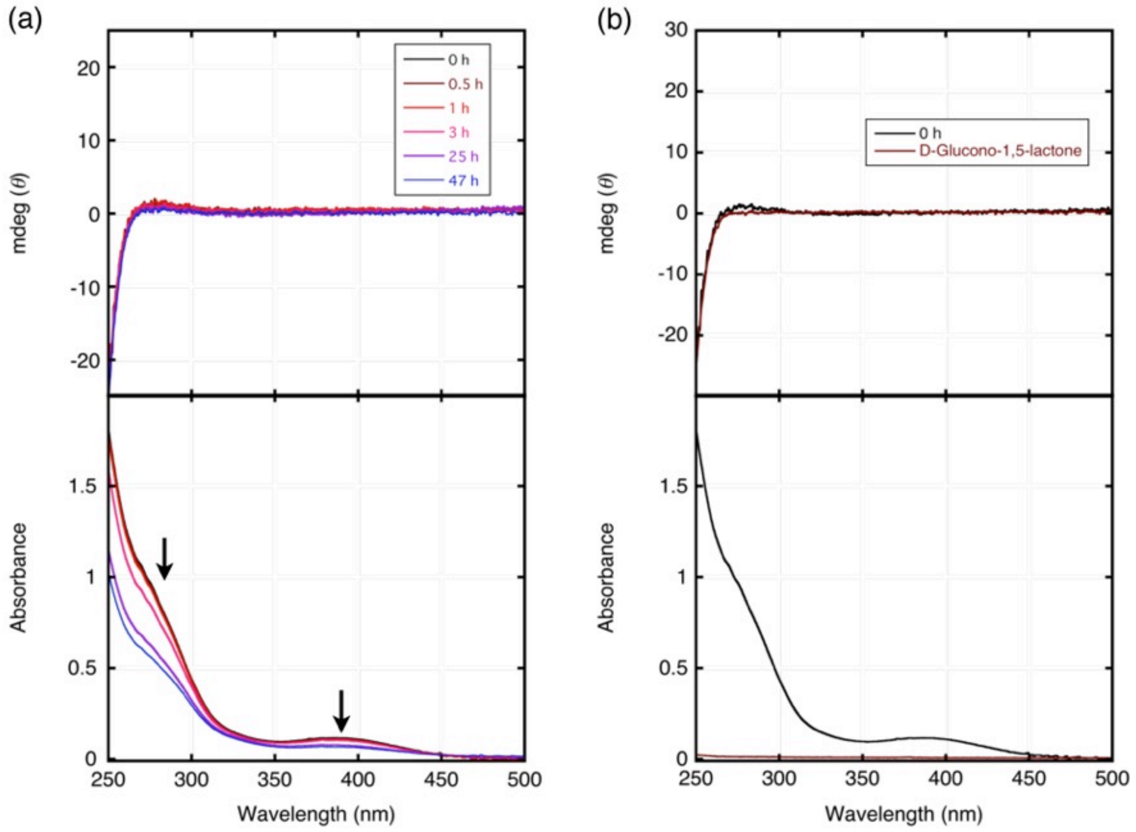

Figure S9. Solution-phase CD and UV-vis absorption spectra of a solution containing ligand **L**, 3 equiv. of $PdCl_2(CH_3CN)_2$, and 30 equiv. of D-glucono-1,5-lactone ([**L**] = 0.8 mM, 25 °C, 1:9 (v/v) DMSO/CH_2Cl_2, l = 0.1 cm). (a) Time course measurements, (b) comparison between the solution of ligand **L**, 3 equiv. of $PdCl_2(CH_3CN)_2$ and 30 equiv. of D-glucono-1,5-lactone and that of D-glucono-1,5-lactone only ([D-glucono-1,5-lactone] = 24 mM, 25 °C, 1:9 (v/v) DMSO/CH_2Cl_2, l = 0.1 cm).

Digestion experiments of as-synthesized (P)- or (M)-enriched MMF-2 into DMSO-d_6

Single-crystals of as-synthesized (*P*)- or (*M*)-enriched MMF-2 were collected and air-dried for 30 sec. The resulting crystals were dissolved in DMSO-d_6 (500 μL), followed by ^{1}H NMR measurement.

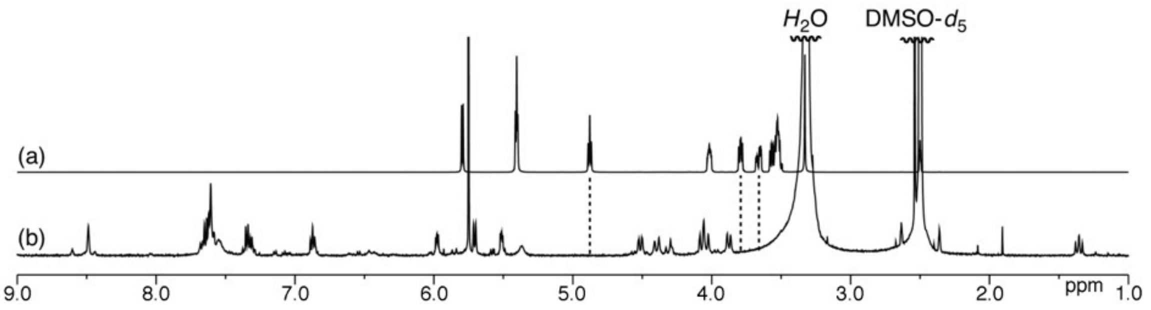

Figure S10. ^{1}H NMR spectra (500 MHz, DMSO-d_6, 300 K) of (a) D-glucurono-6,3-lactone and (b) a digested sample of as-synthesized (*M*)-enriched MMF-2.

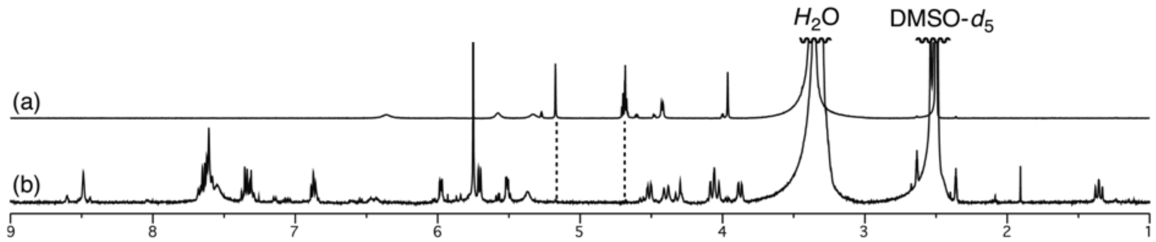

Figure S11. ^{1}H NMR spectra (500 MHz, DMSO-d_6, 300 K) of (a) D-glucono-1,5-lactone and (b) a digested sample of as-synthesized (*P*)-enriched MMF-2.

3. Synthesis and characterization of *rac*-MMF-2

Synthesis of rac-MMF-2

Ligand **L** (3.18 mg, 5.04 μmol) was dissolved in a mixed solvent of CH_2Cl_2 (400 μL) and DMSO (203 μL). To this solution was added a DMSO solution of $PdCl_2(CH_3CN)_2$ (50.0 mM, 301 μL, 15.1 μmol, 3.00 equiv.) and H_2O (5.0 μL). The solution was divided into six equal parts (150 μL each), to which was added CH_2Cl_2 (680 μL). The resulting mixture was then kept standing at 25 °C for a week to obtain yellow block single-crystals (as-synthesized crystals). The yellow crystals were washed with a 1:1 (v/v) mixed solvent of CH_2Cl_2/CH_3CN and soaked in a 1:1 (v/v) mixed solvent of CH_2Cl_2/CH_3CN. After 1 day, the crystals were washed with CH_2Cl_2 and soaked in CH_2Cl_2 for 1 day. The resulting crystals were collected and air-dried quickly on a filter paper to obtain *rac*-MMF-2 single-crystals, $Pd_3\mathbf{L}Cl_6\cdot(CH_2Cl_2)_{5.7}\cdot(H_2O)_{1.5}$, (3.96 mg, 2.37 μmol, 47%). The washed *rac*-MMF-2 crystals were characterized by elemental analysis, IR spectroscopy, thermogravimetric analysis, powder and single-crystal X-ray diffraction (XRD). The

amount of CH_2Cl_2 contained in the single-crystals was determined by ^{1}H NMR spectrum of a digested sample, and the amount of H_2O was calculated by the least square refinement using elemental analysis data shown below.

Elemental analysis: calcd for $C_{42}H_{42}Cl_6N_6Pd_3{\cdot}(CH_2Cl_2)_{5.7}{\cdot}(H_2O)_{1.5}$: C 34.23, H 3.40, N 5.02; found: C 34.40, H 3.78, N 4.83.
IR (ATR): ν 3503, 3092, 2845, 1732, 1716, 1698, 1610, 1558, 1541, 1497, 1456, 1422, 1340, 1267, 1219, 1162, 1113, 1038, 1023, 988, 950, 854 cm^{-1}.

Crystal data for $(Pd_3\mathbf{L}Cl_6)_2{\cdot}(CH_2Cl_2)_{6.09}{\cdot}(H_2O)_{6.18}$: $C_{90.09}H_{108.55}Cl_{24.19}N_{12}O_{6.18}Pd_6$, F_w = 2954.44, crystal dimensions 0.33 × 0.30 × 0.30 mm^3, cubic, space group $I2_13$, a = 31.4049(6) Å, V = 30973.7(10) Å^3, Z = 8, ρ_{calcd} = 1.267 g cm^{-3}, μ = 1.139 cm^{-1}, T = 93 K, λ (MoKα) = 0.71075 Å, $2\theta_{max}$ = 55.0°, 149996/11849 reflections collected/unique, R_1 = 0.0595 ($I > 2\sigma(I)$), wR_2 = 0.1548 (for all data), GOF = 1.089, Flack parameter = 0.50(5) (Friedel Pairs = 5604), largest diff. peak and hole 1.11/–0.64 eÅ^{-3}. CCDC deposit number 989061. See below for the details of applied restraints and response to alert A pointed out in the IUCR's checkCIF routine.

Restraints for dichloromethane molecules
Geometrical restraints; DFIX (d = 1.7800, s = 0.0200): Cl1S–C1S, C1S–Cl2S, Cl1T–C1T, C1T–Cl2T, Cl3S–C2S, Cl4S–C2S, C3S–Cl5S, C3S–Cl6S, C4S–Cl7S. DANG (d = 2.8519 s = 0.0400): Cl1S–Cl2S. DANG (d = 2.7850, s = 0.0400): Cl1T–Cl2T. DANG (d = 2.8756 s = 0.0400): Cl3S–Cl4S. DANG (d = 2.8837, s = 0.0400): Cl6S–Cl5S.

PLAT602_ALERT_2_A Structure contains solvent accessible VOIDS
Response: Several solvents were severely disordered.

Composition analyses of rac-MMF-2 by digestion experiments
The washed *rac*-MMF-2 crystals were collected and air-dried quickly on a filter paper. The crystals were dissolved in DMSO-d_6 (ca. 500 µL). The resulting DMSO-d_6 solution

was analyzed by ^{1}H NMR spectroscopy in the presence of DCl/D_2O ([DCl] = ca. 140 mM).

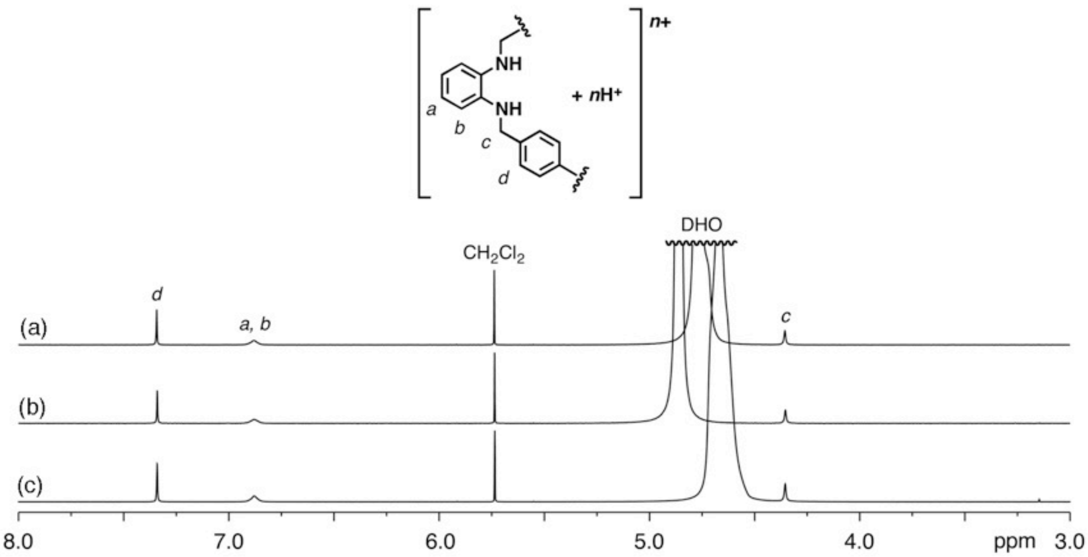

Figure S12. Partial ^{1}H NMR spectra (500 MHz, DMSO-d_6, 293 K) after dissolving crystals in DMSO-d_6 in the presence of excess DCl/D_2O. (a) 1st, (b) 2nd and (c) 3rd trials.

Table S1. Molar ratios of ligand **L** to CH_2Cl_2 determined by ^{1}H NMR spectroscopy

	Molar ratio of ligand to CH_2Cl_2^{a}
1st trial	1 : 5.86
2nd trial	1 : 6.03
3rd trial	1 : 5.42
Average	1 : 5.7(3)

a: The molar ratio was calculated by the integral ratio of signal *d* to CH_2Cl_2.

Table S2. The number of CH_2Cl_2 molecules encapsulated in a unit cell determined by ^{1}H NMR spectroscopy

CH_2Cl_2 in the unit cell	CH_2Cl_2-occupied void space (Å^3, %)
91.2	5563, 34

The unit cell is composed of 16 Pd_3-complexes. The volume of CH_2Cl_2 was calculated on Spartan 06 (61 Å^3) and the equilibrium geometry was determined by DFT calculation

(B3LYP 6-31G*). The total volume of the void space of a unit cell is 16173 Å^3 calculated by PLATON.

Thermogravimetric analysis of rac-MMF-2

The washed *rac*-MMF-2 crystals preserved in CH_2Cl_2 were collected on a filter paper and air-dried quickly. The resulting crystals were analyzed by thermogravimetric analysis.

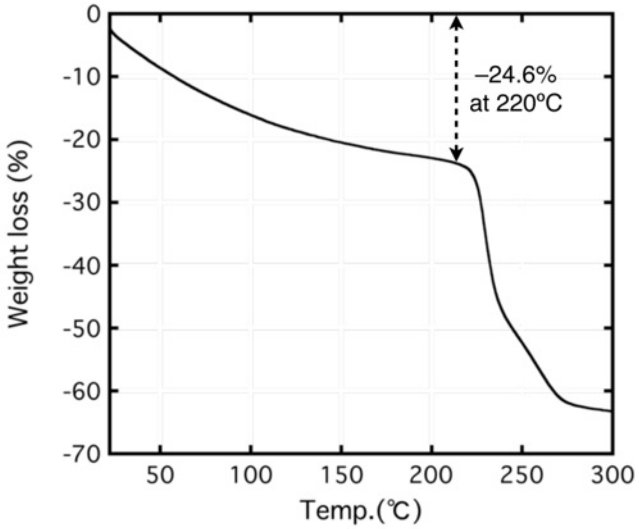

Figure S13. Thermogravimetric analysis of *rac*-MMF-2. Measurement condition: 22 ~ 300 °C, 5 K/min, N_2 flow 150 mL/min. The 25% weight loss was approximately equal to the amount of solvents included in the crystals (31%).

4. Synthesis and characterization of (*M*)-enriched MMF-2

Synthesis of (M)-enriched MMF-2

Ligand **L** (2.87 mg, 4.55 μmol) was dissolved in a mixed solvent of CH_2Cl_2 (360 μL) and DMSO (8 μL). To this solution was added a DMSO solution of D-glucurono-6,3-lactone (1.82 M, 175 μL, 319 μmol, 70 equiv.), a DMSO solution of $PdCl_2(CH_3CN)_2$ (50.1 mM, 272 μL, 13.6 μmol, 3.0 equiv.) and H_2O (4.5 μL). The resulting solution was divided into six equally parts (130 μL each), to which was added CH_2Cl_2 (590 μL). The resulting mixture was then kept standing at 25 °C for a week to obtain yellow block single-crystals (as-synthesized crystals). The yellow crystals were washed with a 1:1 (v/v) mixed solvent of CH_2Cl_2 and CH_3CN and soaked in a 1:1 (v/v)

mixed solvent of CH_2Cl_2 and CH_3CN. After 1 day, the crystals were washed with CH_2Cl_2 and soaked in CH_2Cl_2 for 1 day. The resulting crystals were collected and air-dried quickly on a filter paper to obtain (*M*)-enriched MMF-2 single-crystals, $Pd_3\mathbf{L}Cl_6\cdot(CH_2Cl_2)_{6.9}$, (4.70 mg, 2.69 μmol, 59%). The washed (*M*)-enriched MMF-2 crystals were characterized by elemental analysis and multiple single-crystal XRD. The amount of CH_2Cl_2 contained in a single-crystal was determined by ^{1}H NMR spectroscopy using a digested sample, and the amount of H_2O was calculated by the least square refinement using elemental analysis data shown below.

Elemental analysis: calcd for $C_{42}H_{42}Cl_6N_6Pd_3\cdot(CH_2Cl_2)_{6.9}$: C 33.58, H 3.22, N 4.81; found: C 33.97, H 3.57, N 4.90.

Crystal data for $(Pd_3\mathbf{L}Cl_6)_2\cdot(CH_2Cl_2)_{4.81}\cdot(H_2O)_{5.54}$: $C_{88.81}H_{104.70}Cl_{21.61}N_{12}O_{5.54}Pd_6$, F_w = 2833.48, crystal dimensions 0.32 × 0.26 × 0.25 mm^3, cubic, space group $I2_13$, a = 31.3834(10) Å, V = 30909.9(17) Å^3, Z = 8, ρ_{calcd} = 1.218 g cm^{-3}, μ = 10.951 cm^{-1}, T = 95 K, λ (MoKα) = 0.71075 Å, $2\theta_{max}$ = 55.0°, 142621/11829 reflections collected/unique, R_1 = 0.0840 ($I > 2\sigma(I)$), wR_2 = 0.2381 (for all data), GOF = 1.037, Flack parameter = 0.06(7) (Friedel Pairs = 5594), largest diff. peak and hole 2.02/–0.88 eÅ^{-3}. CCDC deposit number 989063. See below for details of applied restraints and response to alert A pointed out in the IUCR's checkCIF routine.

Restraints for dichloromethane molecules

Geometrical restraints; DFIX (d = 1.7800, s = 0.0200): Cl1S–C1S, Cl1T–C1S, Cl2S–C2S, C2S–Cl2T, Cl3S–C3S, C3S–Cl3T. DANG (d = 2.8900, s = 0.0400): Cl1S–Cl1T. DANG (d = 2.9173, s = 0.0400): Cl2S–Cl2T. DANG (d = 2.9100 s = 0.0400): Cl3S–Cl3T.

PLAT602_ALERT_2_A Structure contains solvent accessible VOIDS

Response: Several solvents were severely disordered.

Composition analyses of (M)-enriched MMF-2 by digestion experiments

The washed (*M*)-enriched MMF-2 crystals were collected and air-dried quickly on a filter paper. The crystals were dissolved in DMSO-d_6 (ca. 500 μL). The resulting DMSO-d_6 solution was analyzed by ^{1}H NMR spectroscopy in the presence of DCl/D_2O ([DCl] = ca. 140 mM).

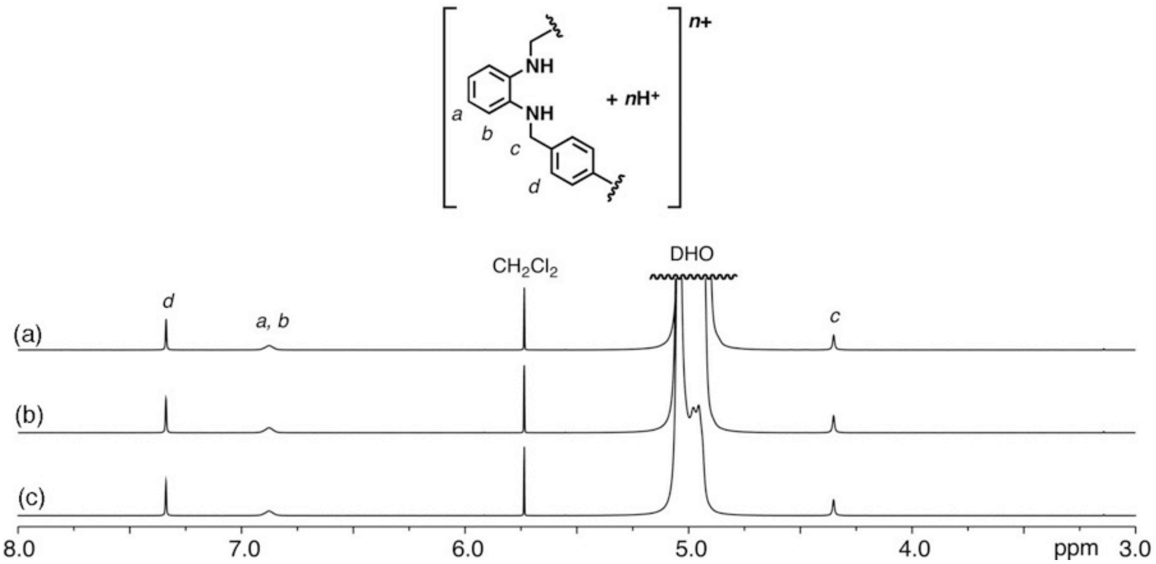

Figure S14. Partial ^{1}H NMR spectra (500 MHz, DMSO-d_6, 293 K) after dissolving crystals in DMSO-d_6 in the presence of excess DCl/D_2O. (a) 1st, (b) 2nd and (c) 3rd trials.

Table S3. Molar ratio of ligand **L** to CH_2Cl_2 determined by ^{1}H NMR spectroscopy

	Molar ratio of ligand to CH_2Cl_2^{a}
1st trial	1 : 6.97
2nd trial	1 : 6.97
3rd trial	1 : 6.90
Average	1 : 6.95(4)

a: The molar ratio was calculated by the integral ratio of signal *d* to CH_2Cl_2.

Table S4. The number of CH_2Cl_2 molecules encapsulated in a unit cell determined by ^1H NMR spectroscopy

CH_2Cl_2 in a unit cell	CH_2Cl_2-occupied void space (Å^3, %)
111	6783, 42

A unit cell is composed of 16 Pd_3-complexes. The volume of CH_2Cl_2 was calculated on Spartan 06 (61 Å^3) and the equilibrium geometry was determined by DFT calculation (B3LYP 6-31G*). The total volume of the void space of a unit cell is 16173 Å^3 calculated by PLATON.

Table S5. Summary of crystal data of seven randomly selected and washed MMF-2 crystals synthesized under an optimized condition

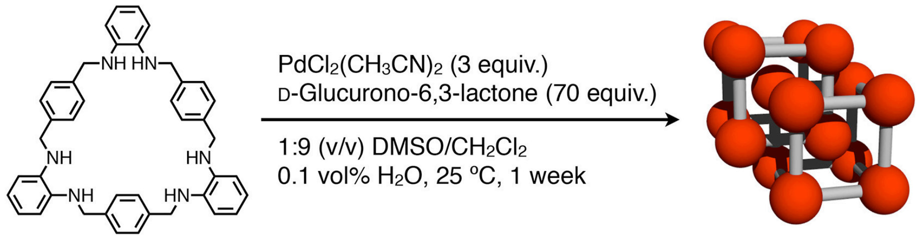

L **(*M*)-enriched MMF-2**

Entry	Crystal size (mm)	*a*	R_1	wR_2	Flack parameter	*P* : *M*	BASF
1	0.32 × 0.26 × 0.25	31.383(1)	0.0840	0.2381	0.06(7)	6:94	6.40
2	0.30 × 0.27 × 0.21	31.3648(7)	0.0884	0.2496	0.06(7)	6:94	6.70
3	0.28 × 0.26 × 0.24	31.436(2)	0.1133	0.3060	0.06(10)	6:94	6.48
4	0.29 × 0.26 × 0.24	31.276(2)	0.1082	0.2945	0.02(9)	2:98	6.46
5	0.29 × 0.24 × 0.22	31.555(4)	0.1198	0.3316	0.02(11)	2:98	4.97
6	0.26 × 0.25 × 0.23	31.4360(7)	0.1394	0.3554	0.07(10)	7:93	5.21
7	0.22 × 0.20 × 0.18	31.5133(8)	0.1106	0.2973	0.03(9)	3:97	8.36

BASF (batch scale factor): ratio (%) of the minor component generated by the rotational twin

Optimization of asymmetric crystallization conditions for (M)-enriched MMF-2

Optimization of the amount of water

Ligand **L** (1.63 mg, 2.58 μmol) was dissolved in a mixed solvent of CH_2Cl_2 (200 μL) and DMSO (56 μL). To this solution were added a DMSO solution of D-glucurono-6,3-lactone (1.34 M, 48 μL, 64.3 μmol, 25 equiv.) and $PdCl_2(CH_3CN)_2$ (50.3 mM, 154 μL, 7.75 μmol, 3.0 equiv.). The resulting solution was divided into 6

portions (70 μL). The appropriate amount of water (0.4 ~ 4 μL) and CH_2Cl_2 (32 μL) were added to each sample, and the resulting solutions were kept standing for a week to afford single-crystals of MMF-2. The single-crystals were washed with and soaked in a 1:1 (v/v) mixed solvent of CH_2Cl_2 and CH_3CN for 1 day, and then washed with and soaked in pure CH_2Cl_2 for 1 day. The resulting washed crystals were analyzed by single-crystal X-ray diffraction.

Optimization of the amount of D-glucurono-6,3-lactone

Ligand **L** (1.26 mg, 2.00 μmol) was dissolved in CH_2Cl_2 (160 μL). To a CH_2Cl_2 solution of ligand **L** were added a DMSO solution of D-glucurono-6,3-lactone (1.80 M, 79 μL, 142 μmol, 71 equiv.), $PdCl_2(CH_3CN)_2$ (49.9 mM, 120 μL, 5.99 μmol, 3.0 equiv.) and water (2.0 μL). This solution was divided into 3 portions (110 μL). CH_2Cl_2 (500 μL) were added to each sample, and the resulting solutions were kept standing for a week to afford single-crystals of MMF-2. The single-crystals were washed with and soaked in a 1:1 (v/v) mixed solvent of CH_2Cl_2 and CH_3CN for 1 day, and then washed with and soaked in pure CH_2Cl_2 for 1 day. The resulting washed crystals were analyzed by single-crystal X-ray diffraction.

Table S6. Optimization of the amount of water for asymmetric crystallization of MMF-2

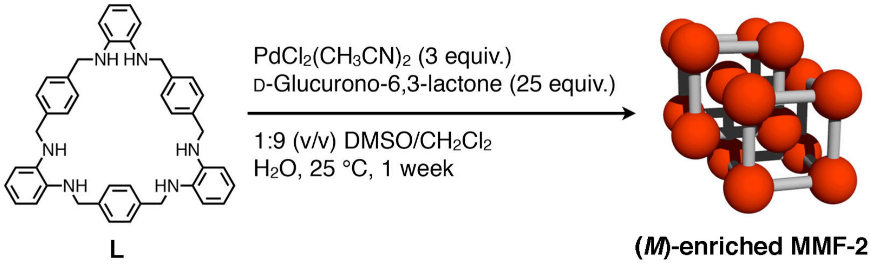

Entry	Equiv. of water	Crystal size (mm)	*a* (Å)	R_1	wR_2	Flack parameter	*P* : *M*	BASF
1	0	0.23 × 0.20 × 0.17	31.3518(8)	0.0823	0.2181	0.10(7)	10:90	19.53
2	0.1	0.32 × 0.29 × 0.19	31.488(2)	0.0794	0.2065	0.27(8)	27:73	30.92
3	0.5	0.47 × 0.37 × 0.32	31.343(2)	0.0812	0.2171	0.37(8)	37:63	40.47
4	1.0	0.47 × 0.40 × 0.32	31.397(1)	0.0728	0.1892	0.28(7)	28:72	35.81

BASF (batch scale factor): ratio (%) of the minor component generated by the rotational twin

Table S7. Optimization of the amount of D-glucurono-6,3-lactone for asymmetric crystallization of MMF-2

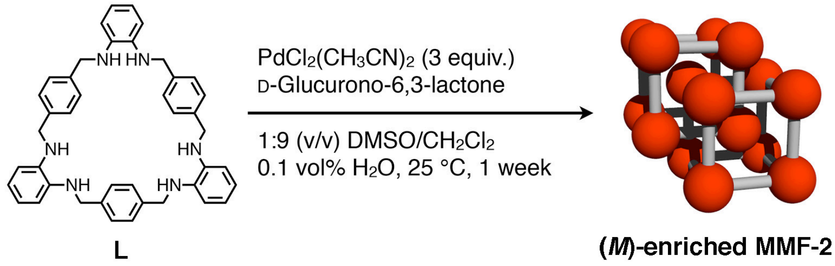

Entry	Equiv. of lactone	Crystal size (mm)	*a*	R_1	wR_2	Flack parameter	*P* : *M*	BASF
1	25	0.32 × 0.29 × 0.19	31.488(2)	0.0794	0.2065	0.27(8)	27:73	30.92
2	40	0.22 × 0.21 × 0.20	31.389(2)	0.0877	0.2267	0.22(9)	22:78	28.92
3	50	0.31 × 0.29 × 0.27	31.4133(8)	0.0672	0.1852	0.09(6)	9:91	15.35
4	60	0.30 × 0.28 × 0.26	31.3995(8)	0.0862	0.2414	0.09(7)	9:91	10.10
5	70	0.32 × 0.26 × 0.25	31.383(1)	0.0840	0.2381	0.06(7)	6:94	6.40

BASF (batch scale factor): ratio (%) of the minor component generated by the rotational twin

5. Synthesis and characterization of (*P*)-enriched MMF-2

Synthesis of (P)-enriched MMF-2

Ligand **L** (3.26 mg, 5.17 μmol) was dissolved in a mixed solvent of CH_2Cl_2 (410 μL) and DMSO (122 μL). To this solution were added a DMSO solution of D-glucono-1,5-lactone (1.80 M, 86 μL, 155 μmol, 30.0 equiv.), a DMSO solution of $PdCl_2(CH_3CN)_2$ (50.2 mM, 309 μL, 15.5 μmol, 3.0 equiv.) and H_2O (5.2 μL). The resulting solution was divided into six equal parts (155 μL each), to which was added CH_2Cl_2 (860 μL). The resulting mixture was then kept standing at 25 °C for a week to obtain yellow block single-crystals (as-synthesized crystals). The yellow crystals were washed with a 1:1 (v/v) mixed solvent of CH_2Cl_2 and CH_3CN and soaked in a 1:1 (v/v) mixed solvent of CH_2Cl_2 and CH_3CN. After 1 day, the crystals were washed with CH_2Cl_2 and soaked in CH_2Cl_2 for 1 day. The resulting crystals were collected and air-dried quickly on a filter paper to obtain single-crystals of (*P*)-enriched MMF-2, $Pd_3\mathbf{L}Cl_6\cdot(CH_2Cl_2)_6\cdot(H_2O)$, (3.12 mg, 1.85 μmol, 36%). The washed (*P*)-enriched MMF-2 crystals were characterized by elemental analysis and multiple single-crystal XRD. The amount of CH_2Cl_2 contained in a single-crystal was determined by ^1H NMR spectroscopy

using a digested sample, and the amount of H_2O was calculated by the least square refinement using elemental analysis data shown below.

Elemental analysis: calcd for $C_{42}H_{42}Cl_6N_6Pd_3\cdot(CH_2Cl_2)_{6.0}\cdot(H_2O)$: C 34.11, H 3.34, N 4.97; found: C 34.54, H 3.57, N 4.41.

Crystal data for $(Pd_3\mathbf{L}Cl_6)_2\cdot(CH_2Cl_2)_{4.77}\cdot(H_2O)_{4.92}$: $C_{88.77}H_{103.38}Cl_{21.54}N_{12}O_{4.92}Pd_6$, F_w = 2819.27, crystal dimensions 0.25 × 0.23 × 0.21 mm^3, cubic, space group $I2_13$, a = 31.4950(7) Å, V = 31240.9(12) Å^3, Z = 8, ρ_{calcd} = 1.199 g cm^{-3}, μ = 10.817 cm^{-1}, T = 97 K, λ (MoKα) = 0.71075 Å, $2\theta_{max}$ = 55.0°, 136000/11931 reflections collected/unique, R_1 = 0.0917 ($I > 2\sigma(I)$), wR_2 = 0.2429 (for all data), GOF = 1.045, Flack parameter = 0.07(8) (Friedel Pairs = 5643), largest diff. peak and hole 1.66 /–0.64 eÅ^{-3}. CCDC deposit number 989062. See below for the details of applied restraints and response to alert A pointed out in the IUCR's checkCIF routine.

Restraints for dichloromethane molecules

Geometrical restraints; DFIX (d = 1.7800, s = 0.0100): Cl1S–C1S, C1S–Cl1T, Cl2S–C2S, C2S–Cl2T, Cl3S–C3S, C3S–Cl3T. DANG (d = 2.9028, s = 0.0200): Cl1S–Cl1T, DANG (d = 2.8996, s = 0.0200): Cl2S–Cl2T, DANG (d = 2.9025, s = 0.0400): Cl3S–Cl3T.

PLAT602_ALERT_2_A Structure contains solvent accessible VOIDS

Response: Several solvents were severely disordered.

Table S8. Summary of crystal data of six randomly selected MMF-2 crystals synthesized under an optimized condition

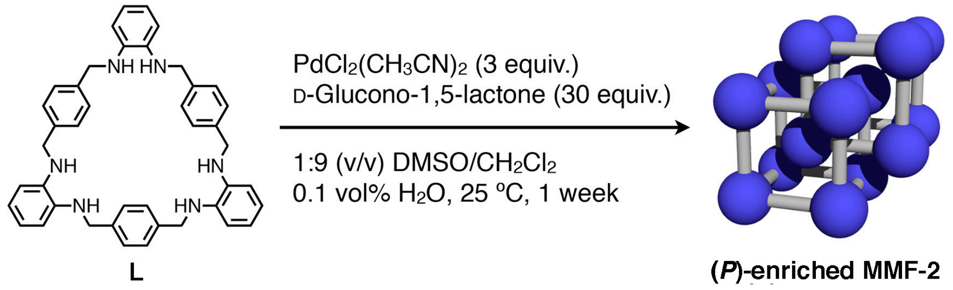

Entry	Crystal size (mm)	*a* (Å)	R_1	wR_2	Flack parameter	*P* : *M*	BASF
1	0.41 × 0.37 × 0.26	31.3632(7)	0.0984	0.2726	0.07(9)	93:7	19.13
2	0.35 × 0.31 × 0.29	31.3420(6)	0.1528	0.3705	0.09(13)	91:9	23.03
3	0.25 × 0.23 × 0.21	31.4950(7)	0.0917	0.2429	0.07(8)	93:7	17.50
4	0.21 × 0.19 × 0.18	31.4509(6)	0.0850	0.2273	0.16(8)	84:16	21.88
5	0.23 × 0.21 × 0.20	31.5257(9)	0.0999	0.2627	0.04(9)	96:4	18.19
6	0.21 × 0.18 × 0.16	31.5590(6)	0.0895	0.2339	0.15(8)	85:15	23.12

BASF (batch scale factor): ratio (%) of the minor component generated by the rotational twin

Optimization of asymmetric crystallization condition for (P)-enriched MMF-2

Optimization of the amount of D*-glucono-1,5-lactone*

Ligand **L** (1.10 mg, 1.74 μmol) was dissolved in a mixed solvent of CH_2Cl_2 (140 μL) and DMSO (42 μL). To a DMSO-CH_2Cl_2 solution of ligand **L** were added a DMSO solution of D-glucono-1,5-lactone (1.83 M, 28.6 μL, 52.3 μmol, 30 equiv.), $PdCl_2(CH_3CN)_2$ (50.2 mM, 104 μL, 5.22 μmol, 3.0 equiv.) and H_2O (1.7 μL). This solution was divided into 3 portions (90 μL). CH_2Cl_2 (410 μL) was added to each sample, and the resulting solutions were kept for a week to afford single-crystals of MMF-2. The single-crystals were washed with and soaked in a 1:1 (v/v) mixed solvent of CH_2Cl_2 and CH_3CN for 1 day, and then washed with and soaked in pure CH_2Cl_2 for 1 day. The resulting washed crystals were analyzed by single-crystal X-ray diffraction.

Table S9. Optimization of the amount of D-glucono-1,5-lactone for asymmetric crystallization of MMF-2

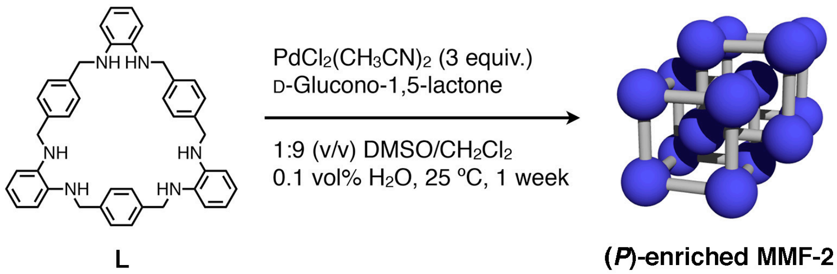

Entry	Equiv. of lactone	Crystal size (mm)	*a*	R_1	wR_2	Flack parameter	*P* : *M*	BASF
1[a]	20	—	—	—	—	—	—	—
2	30	0.41 × 0.37 × 0.26	31.3632(7)	0.0984	0.2726	0.07(9)	7:93	19.13
3	40	0.24 × 0.23 × 0.19	31.4212(6)	0.0785	0.2125	0.14(7)	14:86	20.80

a: Only powder product was obtained.

BASF (batch scale factor): ratio (%) of the minor component generated by the rotational twin

Solid-phase CD measurement of (M)-enriched MMF-2

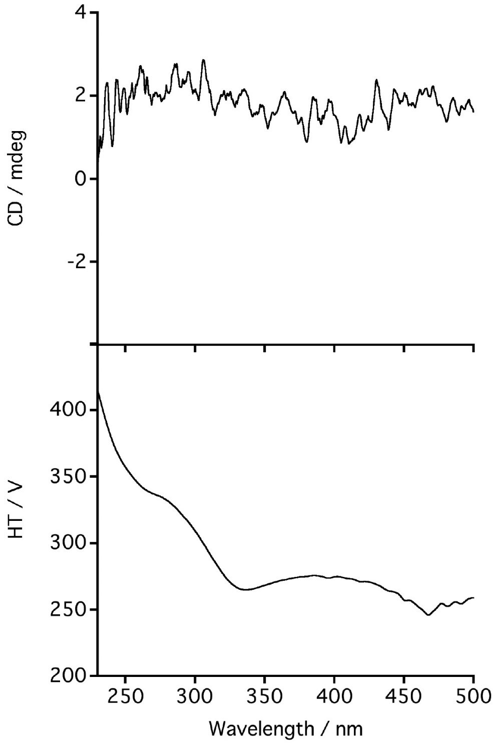

Figure S15. Diffuse reflectance CD (upper) and UV (bottom) spectra of (*M*)-enriched MMF-2 crystals grinded on an agate mortar with KCl.

6. Asymmetric crystallization of MMF-2 using other enantiopure compounds

Asymmetric crystallization using D-ribono-1,4-lactone as a chirality induction reagent

Ligand **L** (1.02 mg, 1.62 μmol) was dissolved in a mixed solvent of CH_2Cl_2 (127 μL) and DMSO (29 μL). To this solution were added a DMSO solution of D-ribono-1,4-lactone (1.83 M, 35.5 μL, 65.0 μmol, 40 equiv.), a DMSO solution of $PdCl_2(CH_3CN)_2$ (50.1 mM, 97 μL, 4.86 μmol, 3.0 equiv.) and H_2O (1.6 μL). The resulting solution was divided into three equal parts (90 μL each). CH_2Cl_2 (410 μL) was added to each sample, and the resulting solutions were kept standing for a week at 25 °C to obtain yellow block single-crystals. The yellow crystals were washed with and soaked in a 1:1 (v/v) mixed solvent of CH_2Cl_2 and CH_3CN for 1 day, then washed with CH_2Cl_2 and soaked in CH_2Cl_2 for 1 day. The washed crystals were analyzed by single-crystal X-ray diffraction.

Crystal data for $(Pd_3\mathbf{L}Cl_6)_2\cdot(CH_2Cl_2)_{4.28}\cdot(H_2O)_{4.89}$: $C_{88.28}H_{102.34}Cl_{20.56}N_{12}O_{4.89}Pd_6$, F_w = 2777.11, crystal dimensions 0.26 × 0.24 × 0.22 mm^3, cubic, space group $I2_13$, a = 31.4527(16) Å, V = 31115(3) Å^3, Z = 8, ρ_{calcd} = 1.186 g cm^{-3}, μ = 10.689 cm^{-1}, T = 100 K, λ (MoKα) = 0.71075 Å, $2\theta_{max}$ = 55.0°, 137623/11870 reflections collected/unique, R_1 = 0.0940 ($I > 2\sigma(I)$), wR_2 = 0.2416 (for all data), GOF = 1.030, Flack parameter = 0.25(9) (Friedel Pairs = 5610), largest diff. peak and hole 3.43 /–1.01 eÅ^{-3}. CCDC deposit number 989064. See below for the details of applied restraints and response to alert A pointed out in the IUCR's checkCIF routine.

Restraints for dichloromethane molecules

Geometrical restraints; DFIX (d = 1.7800, s = 0.0100): Cl1S–C1S, Cl1T–C1S, Cl2S–C2T, Cl2T–C2T, Cl3S–C3S, Cl3T–C3S. DANG (d = 2.8842, s = 0.0200): Cl1S–Cl1T. DANG (d = 2.8924, s = 0.0200) Cl2S–Cl2T. DANG (d = 2.9132, s = 0.0400) Cl3S–Cl3T.

PLAT602_ALERT_2_A Structure contains solvent accessible VOIDS

Response: Several solvents were severely disordered.

Asymmetric crystallization using L-gulonic acid γ-lactone as a chirality induction reagent

Ligand **L** (0.62 mg, 0.98 μmol) was dissolved in a mixed solvent of CH_2Cl_2 (85 μL) and DMSO (19 μL). To this solution were added a DMSO solution of L-gulonic acid γ-lactone (1.04 M, 17 μL, 17.7 μmol, 18 equiv.) and a DMSO solution of $PdCl_2(CH_3CN)_2$ (47.9 mM, 62 μL, 2.97 μmol, 3.0 equiv.). To this solution was added CH_2Cl_2 (800 μL), and the resulting solution was kept standing for a week at 25 °C to obtain yellow block single-crystals. The yellow crystals were washed with a 1:1 (v/v) mixed solvent of CH_2Cl_2 and CH_3CN and soaked in a 1:1 (v/v) mixed solvent of CH_2Cl_2 and CH_3CN for 1 day, then washed with and soaked in CH_2Cl_2 for 1 day. The washed crystals were analyzed by single-crystal X-ray diffraction.

Crystal data for $(Pd_3\mathbf{L}Cl_6)_2{\cdot}(CH_2Cl_2)_{4.82}{\cdot}(H_2O)_{6.52}$: $C_{88.82}H_{106.67}Cl_{21.63}N_{12}O_{6.52}Pd_6$, F_w = 2851.97, crystal dimensions 0.25 × 0.20 × 0.15 mm^3, cubic, space group $I2_13$, a = 31.493(3) Å, V = 31236(4) Å^3, Z = 8, ρ_{calcd} = 1.213 g cm^{-3}, μ = 10.849 cm^{-1}, T = 93 K, λ (MoKα) = 0.71075 Å, $2\theta_{max}$ = 55.0°, 154203/11897 reflections collected/unique, R_1 = 0.0829 ($I > 2\sigma(I)$), wR_2 = 0.2137 (for all data), GOF = 1.047, Flack parameter = 0.17(8) (Friedel Pairs = 5623), largest diff. peak and hole 1.41/–0.67 eÅ^{-3}. CCDC deposit number 989065. See below for the details of applied restraints and response to alert A pointed out in the IUCR's checkCIF routine.

Restraints for dichloromethane molecules

Geometrical restraints; DFIX (d = 1.7800, s = 0.0100): C1S–Cl1S, C1S–Cl1T, C2S–Cl2T, Cl2S–C2S, C3S–Cl3S, Cl3T–C3S. DANG (d = 2.8931, s = 0.0200): Cl1T–Cl1S. DANG (d = 2.8846, s = 0.0200): Cl2T–Cl2S. DANG (d = 2.8753, s = 0.0400): Cl3T–Cl3S

PLAT602_ALERT_2_A Structure contains solvent accessible VOIDS

Response: Several solvents were severely disordered.

PLAT430_ ALERT_2_A Short Inter D...A Contact O4W .. O4W .. 2.51 Ang.

Response: A water molecule was disordered along a three rotational axis. ;

General procedure of trials of asymmetric crystallization using various kinds of optically-pure compounds

Ligand **L** (0.36 mg, 0.57 μmol) was dissolved in CH_2Cl_2 (71 μL). To this solution were added a saturated DMSO solution of L-phenylalanine (21 μL) and a DMSO solution of $PdCl_2(CH_3CN)_2$ (47.9 mM, 36 μL, 1.72 μmol, 3.0 equiv.). CH_2Cl_2 (500 μL) was added to the solution, and the resulting mixture was kept standing for a week at 25 °C to obtain yellow block single-crystals. The yellow crystals were washed with a 1:1 (v/v) mixed solvent of CH_2Cl_2 and CH_3CN and soaked in a 1:1 (v/v) mixed solvent of CH_2Cl_2 and CH_3CN for 1 day, then washed with and soaked in CH_2Cl_2 for 1 day. The washed crystals were analyzed by single-crystal X-ray diffraction.

Table S10. Summary for asymmetric crystallization in the presence of chiral additives

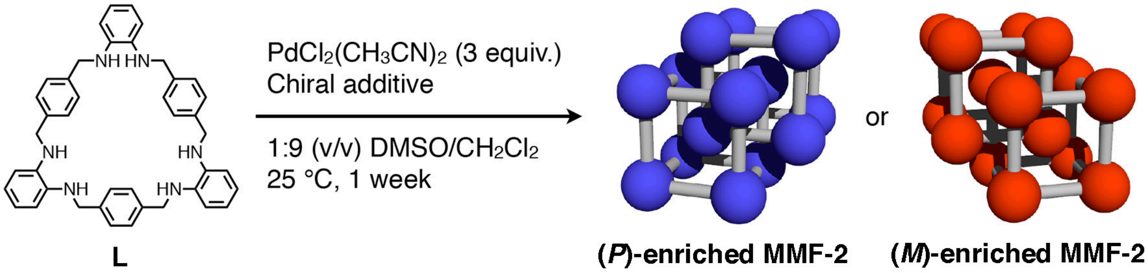

Entry	Chiral additive	Equiv.	Results	Flack parameter	*P* : *M*
1	D-Glucose	12.5	Amorphous	–	–
2	D-Mannose	12.6	Amorphous	–	–
3	Sucrose	3.1	Single crystals	0.31(10)	69:31
4	L-Tartaric acid	9.4	No solid	–	–
5	(–)-Menthol	100	Single crystals	0.34(17)	34:66
6	Cinchonidine	10	No solid	–	–
7	Camphor	1000	Single crystals	0.45(8)	55:45
8	(*R*)-Propylene carbonate	1000	Yellow powder	–	–
9	(–)-2,3-*O*-Isopropylidene-D-threitol	1000	Yellow powder	–	–
10	L-Phenylalanine	–[a]	Single crystals	0.22(7)	22:78
11	Phe-OMe•HCl salt	–[a]	No solid	–	–
12	L-Tyrosine	–[a]	No solid	–	–
13	L-Histidine	–[a]	Single crystals	0.44(11)	44:56
14	L-Tryptophane	–[a]	No solid	–	–
15	L-Arginine	–[a]	Yellow powder	–	–
16	L-Proline	–[a]	Single crystals	0.43(10)	43:57
17	Stirring[b]	–	Yellow powder	–	–

a: Saturated DMSO solutions of chiral additives were used. *b*: The solution was stirred during crystallization.

7. Crystal growth experiments

Crystal growth experiment of (M)-enriched MMF-2 as a seed crystal

A washed (*M*)-enriched MMF-2 crystal was analyzed by single-crystal X-ray diffraction. The analyzed crystal was used as a seed crystal. A supersaturated solution of $Pd_3\mathbf{L}Cl_6$ in 1:9 (v/v) DMSO/CH_2Cl_2 was prepared as follows. Ligand **L** (0.58 mg, 0.92 μmol) was dissolved in a mixed solvent of CH_2Cl_2 (67 μL) and DMSO (37 μL). To this solution were added a DMSO solution of $PdCl_2(CH_3CN)_2$ (50.0 mM, 55 μL, 2.75 μmol, 3.0 equiv.) and CH_2Cl_2 (760 μL). To this supersaturated solution of $Pd_3\mathbf{L}Cl_6$, a seed crystal was added. After a week, the grown crystal was washed with and soaked in a 1:1 (v/v) mixed solvent of CH_2Cl_2 and CH_3CN for 1 day. The crystal was washed with pure CH_2Cl_2 (0.5 mL × 3) and soaked in CH_2Cl_2 for 1 day. The resulting crystal was analyzed by single-crystal X-ray diffraction.

Crystal data of the seed crystal

Crystal data for $(Pd_3\mathbf{L}Cl_6)_2\cdot(CH_2Cl_2)_{4.53}\cdot(H_2O)_{6.96}$: $C_{88.53}H_{106.99}Cl_{21.07}N_{12}O_{6.96}Pd_6$, F_w = 2836.00, crystal dimensions 0.19 × 0.17 × 0.15 mm^3, cubic, space group $I2_13$, a = 31.3922(6) Å, V = 30936.0(10) Å^3, Z = 8, ρ_{calcd} = 1.218 g cm^{-3}, μ = 10.859 cm^{-1}, T = 93 K, λ (MoKα) = 0.71075 Å, $2\theta_{max}$ = 55.0°, 146665/11810 reflections collected/unique, R_1 = 0.0961 ($I > 2\sigma(I)$), wR_2 = 0.2535 (for all data), GOF = 1.024, Flack parameter = 0.20(9) (Friedel Pairs = 5580), largest diff. peak and hole 2.99/–0.83 eÅ^{-3}. CCDC deposit number 989067. See below for the details of applied restraints and response to alert A pointed out in the IUCR's checkCIF routine.

Restraints for dichloromethane molecules

Geometrical restraints; DFIX (d = 1.7800, s = 0.0100): C1S–Cl1S, C1S–Cl1T, C2S–Cl2S, Cl2T–C2S, C3S–Cl3S, Cl3T–C3S. DANG (d = 2.8961, s = 0.0200) Cl1T–Cl1S. DANG (d = 2.8909, s = 0.0200): Cl2S–Cl2T. DANG (d = 2.8874, s = 0.0400): Cl3T–Cl3S.

PLAT602_ALERT_2_A Structure contains solvent accessible VOIDS

Response: Several solvents were severely disordered.

PLAT430_ALERT_2_A Short Inter D...A Contact O3W..O3W..2.30 Ang.

Response: A water molecule was disordered along a three rotational axis.

Crystal data of the grown crystal

Crystal data for $(Pd_3\mathbf{L}Cl_6)_2\cdot(CH_2Cl_2)_{4.34}\cdot(H_2O)_{5.43}$: $C_{88.34}H_{103.54}Cl_{20.68}N_{12}O_{5.43}Pd_6$, F_w = 2791.93, crystal dimensions 0.31 × 0.27 × 0.20 mm^3, cubic, space group $I2_13$, a = 31.3469(8) Å, V = 30802.2(13) Å^3, Z = 8, ρ_{calcd} = 1.204 g cm^{-3}, μ = 10.823 cm^{-1}, T = 93 K, λ (MoKα) = 0.71075 Å, $2\theta_{max}$ = 54.9°, 142347/11787 reflections collected/unique, R_1 = 0.0799 ($I > 2\sigma(I)$), wR_2 = 0.2120 (for all data), GOF = 1.073, Flack parameter = 0.46(8) (Friedel Pairs = 5574), largest diff. peak and hole 1.78/–0.80 eÅ^{-3}. CCDC deposit number 989068. See below for the details of applied restraints and response to alert A pointed out in the IUCR's checkCIF routine.

Restraints for dichloromethane molecules

Geometrical restraints; DFIX (d = 1.7800, s = 0.0100): C1S–Cl1S, C1S–Cl1T, Cl2S–C2S, C2S–Cl2T, C3S–Cl3S, Cl3T–C3S. DANG (d = 2.8990, s = 0.0200): Cl1T–Cl1S. DANG (d = 2.8985, s = 0.0200): Cl2S–Cl2T. DANG (d = 2.8850, s = 0.0200): Cl3S–Cl3T.

PLAT602_ALERT_2_A Structure contains solvent accessible VOIDS

Response: Several solvents were severely disordered.

PLAT430_ALERT_2_A Short Inter D...A Contact O3W..O3W..2.27 Ang.

Response: A water molecule was disordered along a three rotational axis.

Crystal growth experiment of rac-MMF-2 as a seed crystal

A washed *rac*-MMF-2 crystal was analyzed by single-crystal X-ray diffraction. The analyzed crystal was used as a seed crystal. A supersaturated solution of $Pd_3\mathbf{L}Cl_6$ in 1:9 (v/v) DMSO/CH_2Cl_2 was prepared as follows. Ligand **L** (2.95 mg, 4.68 μmol) was dissolved in a mixed solvent of CH_2Cl_2 (400 μL) and DMSO (100 μL). To this solution were added a DMSO solution of D-glucurono-6,3-lactone (1.33 M, 88 μL, 117 μmol, 25.0 equiv.) and a DMSO solution of $PdCl_2(CH_3CN)_2$ (50.1 mM, 280 μL, 14.0 μmol, 3.0 equiv.) (Final concentration: 5.39 mM, 868 μL). The resulting solution (140 μL) and CH_2Cl_2 (760 μL) were mixed to obtain a supersaturated solution of $Pd_3\mathbf{L}Cl_6$. To this

supersaturated solution of $Pd_3\mathbf{L}Cl_6$ a seed crystal was added. After a week, the grown crystals were washed with a 1:1 (v/v) mixed solvent of CH_2Cl_2 and CH_3CN and soaked in a 1:1 (v/v) mixed solvent of CH_2Cl_2 and CH_3CN for 1 day. The crystal was washed with pure CH_2Cl_2 (0.5 mL × 3) and soaked in CH_2Cl_2 for 1 day. The resulting crystal was analyzed by single-crystal X-ray diffraction.

Crystal data of the seed crystal

Crystal data for $(Pd_3\mathbf{L}Cl_6)_2\cdot(CH_2Cl_2)_{4.49}\cdot(H_2O)_{5.10}$: $C_{88.49}H_{103.18}Cl_{20.98}N_{12}O_{5.10}Pd_6$, F_w = 2798.73, crystal dimensions 0.23 × 0.21 × 0.17 mm^3, cubic, space group $I2_13$, a = 31.4043(6) Å , V = 30971.8(10) Å^3, Z = 8, ρ_{calcd} = 1.200 g cm^{-3}, μ = 10.809 cm^{-1}, T = 93 K, λ (MoKα) = 0.71075 Å, $2\theta_{max}$ = 54.9°, 129995/11841 reflections collected/unique, R_1 = 0.0835 ($I > 2\sigma(I)$), wR_2 = 0.2102 (for all data), GOF = 1.016, Flack parameter = 0.41(8) (Friedel Pairs = 5598), largest diff. peak and hole 1.22/–0.82 eÅ^{-3}. CCDC deposit number 989069. See below for the details of applied restraints and response to alert A pointed out in the IUCR's checkCIF routine.

Restraints for dichloromethane molecules

Geometrical restraints; DFIX (d = 1.7800, s = 0.0100): C1S–Cl1S, Cl1T–C1S, Cl2S–C2S, Cl2T–C2S, C3S–Cl3S, Cl3T–C3S. DANG (d = 2.8933, s = 0.0200): Cl1T–Cl1S. DANG (d = 2.8928, s = 0.0200): Cl2S–Cl2T. DANG (d = 2.8878, s = 0.0400): Cl3S–Cl3T.

Restraints on anisotropic displacement parameters; SIMU (s = 0.02 st = 0.04): C1S–Cl1S–Cl1T.

PLAT602_ALERT_2_A Structure contains solvent accessible VOIDS

Response: Several solvents were severely disordered.

PLAT430_ALERT_2_A Short Inter D...A Contact O3W..O3W..2.43 Ang.

Response: A water molecule was disordered along a three rotational axis.

Crystal data of the grown crystal

Crystal data for $(Pd_3\mathbf{L}Cl_6)_2\cdot(CH_2Cl_2)_{4.52}\cdot(H_2O)_{5.14}$: $C_{88.52}H_{103.31}Cl_{21.04}N_{12}O_{5.14}Pd_6$, F_w = 2801.99, crystal dimensions 0.31 × 0.24 × 0.22 mm^3, cubic, space group $I2_13$, a =

31.4477(6) Å, V = 31100.4(10) Å^3, Z = 8, ρ_{calcd} = 1.197 g cm^{-3}, μ = 10.780 cm^{-1}, T = 93 K, λ (MoKα) = 0.71075 Å, 2θ_{max} = 55.0°, 135501/11895 reflections collected/unique, R_1 = 0.0758 ($I > 2\sigma(I)$), wR_2 = 0.1999 (for all data), GOF = 1.070, Flack parameter = 0.19(7) (Friedel Pairs = 5625), largest diff. peak and hole 1.48/–0.80 eÅ^{-3}. CCDC deposit number 989070. See below for the details of applied restraints and response to alert A pointed out in the IUCR's checkCIF routine.

Restraints for dichloromethane molecules

Geometrical restraints; DFIX (d = 1.7800, s = 0.0100): Cl1S–C1S, C1S–Cl1T, Cl2S–C2S, C2S–Cl2T, Cl3S–C3S, C3S–Cl3T, Cl1S–Cl1T. DANG (d = 2.9005, s = 0.0200): Cl2T–Cl2S. DANG (d = 2.8872, s = 0.0200): Cl3T–Cl3S.

PLAT602_ALERT_2_A Structure contains solvent accessible VOIDS

Response: Several solvents were severely disordered.

Table S11. Summary of crystal data before and after further crystal growth

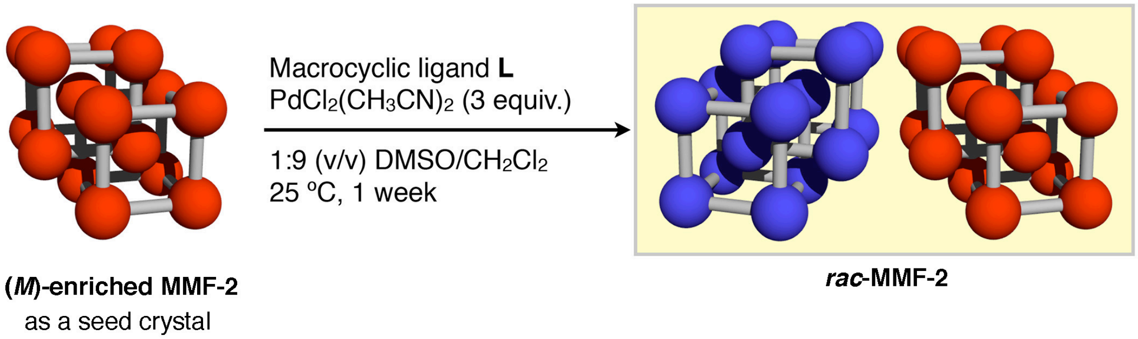

	Crystal size (mm)	R_1	wR_2	Flack parameter	$P:M$
Before growth	0.19 × 0.17 × 0.15	0.0987	0.2619	0.20(10)	20:80
After growth	0.32 × 0.27 × 0.20	0.0833	0.2242	0.46(9)	46:54

Table S12. Summary of crystal data before and after further crystal growth

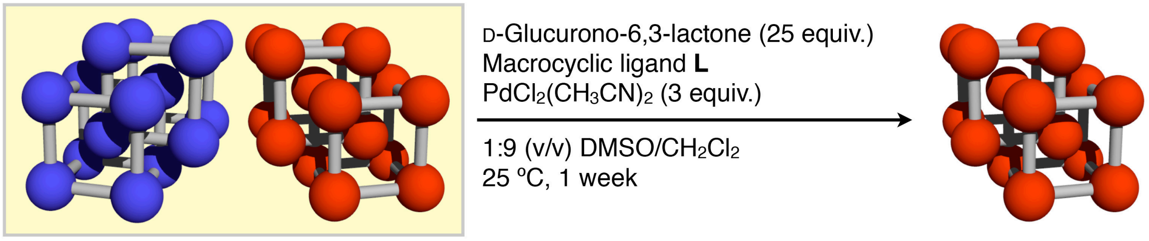

***rac*-MMF-2**
as a seed crystal

(*M*)-enriched MMF-2

	Crystal size (mm)	R_1	wR_2	Flack parameter	*P* : *M*
Before growth	0.23 × 0.21 × 0.17	0.0883	0.2271	0.39(9)	39:61
After growth	0.31 × 0.24 × 0.22	0.0805	0.2180	0.20(8)	20:80

Comment: To evaluate the possibility of chirality induction in the crystal growth process, we conducted crystal growth experiments using a seed crystal of (*M*)-enriched MMF-2 or *rac*-MMF-2 in the absence or presence of an optically-pure lactone, respectively. The size and Flack parameter of the seed crystal of moderately (*M*)-enriched MMF-2 were determined by single-crystal XRD before the crystal growth experiment (crystal size: $0.19 \times 0.17 \times 0.15$ mm^3, Flack parameter: 0.20(9)). The seed crystal was then soaked into a 1:9 (v/v) mixed solvent of DMSO and CH_2Cl_2 solution containing $Pd_3\mathbf{L}Cl_6$ only at 25 °C for a week. The result of single-crystal XRD analysis was that the crystal was significantly grown ($0.31 \times 0.27 \times 0.20$ mm^3), and its Flack parameter approached almost 0.50 (0.46(8)). This result suggests that (*M*)-selective crystal growth did not occur in the outer shell of the resulting single-crystal. In contrast, the crystal growth of a seed crystal of *rac*-MMF-2 (crystal size: $0.23 \times 0.21 \times 0.17$ mm^3, Flack parameter: 0.41(8)) in the presence of D-glucurono-6,3-lactone gave rise to chirality induction. After the crystal growth under the same condition, the crystal was significantly grown ($0.31 \times 0.24 \times 0.22$ mm^3) to an (*M*)-enriched single-crystal (Flack parameter: 0.19(7)). This enantio preference of the crystal growth was the same as expected from that of single-crystals obtained from a solution containing a mixture of (*M*)- and (*P*)-isomers of *syn*-$Pd_3\mathbf{L}Cl_6$ in the presence of D-glucurono-6,3-lactone. This observation suggests that the crystal was grown in an enantioselective manner with the aid of the lactones. Another point of importance is that the chirality induction occurs independently of the crystal surface

features of MMF-2. The involvement of the nucleation process in the chirality induction remains unclear.

8. Synthesis and characterization of MMF-3

Synthesis of MMF-3 through single-crystal to single-crystal transformation

The washed MMF-2 crystals were soaked into $CHCl_3$ at 50 °C for a week. The resulting crystal transformed into MMF-3 through helicity inversion of Pd_3-macrocycles.

Crystal data for $(Pd_3\boldsymbol{L}Cl_6)\cdot(CHCl_3)_{2.94}\cdot(H_2O)$: $C_{47.41}H_{49.41}Cl_{22.23}N_6OPd_3$, F_w = 1826.60, crystal dimensions 0.28 × 0.25 × 0.21 mm^3, cubic, space group *I*-43*d*, *a* = 31.3274(6) Å, V = 30745.0(10) Å^3, Z = 16, ρ_{calcd} = 1.578 g cm^{-3}, μ = 1.502 cm^{-1}, T = 97 K, λ (MoKα) = 0.71075 Å, $2\theta_{max}$ = 54.9°, 143862/5866 reflections collected/unique, R_1 = 0.0629 ($I > 2\sigma(I)$), wR_2 = 0.1823 (for all data), GOF = 1.058, Flack parameter (Friedel Pairs = 2771) = 0.01(6), largest diff. peak and hole 1.40/–1.05 eÅ^{-3}. CCDC deposit number 989066. See below for the details of applied restraints and response to alert A pointed out in the IUCR's checkCIF routine.

Restraints for chloroform molecules

Geometrical restraints; DFIX (*d* = 1.7800, *s* = 0.0200): C3S–Cl7S, C3S–Cl5S, C3S–Cl6S, C2S–Cl4S, C2T–Cl4S, Cl7S–Cl6S. DANG (*d* = 2.8405, *s* = 0.0400): Cl6S–Cl5S. DANG (*d* = 2.8560, *s* = 0.0400): Cl5S–Cl7S.

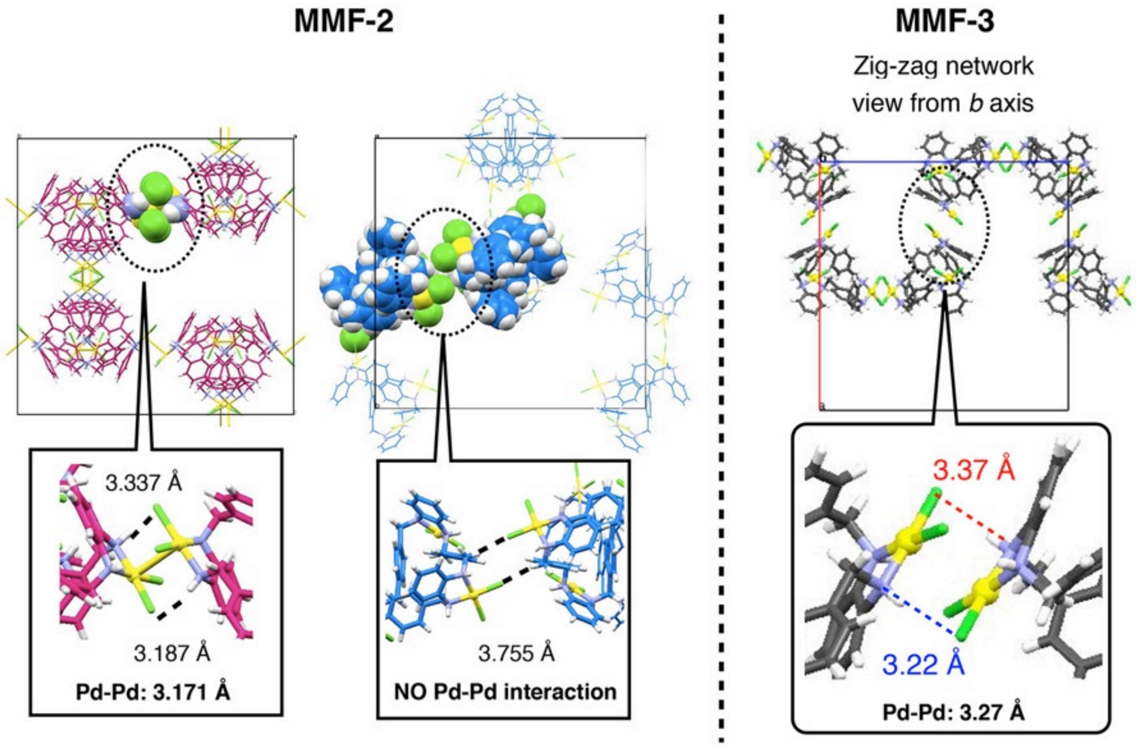

Figure S16. Intermolecular interactions of $Pd_3\mathbf{L}Cl_6$ in MMF-2 (left) and MMF-3 (right).

Composition analyses of MMF-3 by digestion experiments

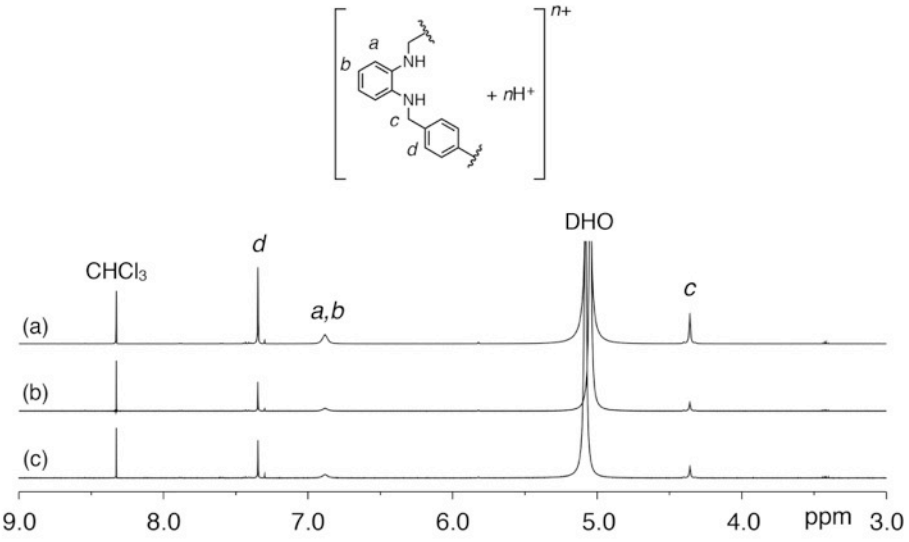

Figure S17. Partial ^{1}H NMR spectra (500 MHz, DMSO-d_6, 293 K) after dissolving crystals in DMSO-d_6 in the presence of excess DCl-D_2O. (a) 1st, (b) 2nd and (c) 3rd trials.

Table S13. Molar ratios of ligand **L** to $CHCl_3$ determined by ^{1}H NMR spectroscopy

	Molar ratio of ligand to $CHCl_3$[a]
1st trial	1 : 4.26
2nd trial	1 : 4.28
3rd trial	1 : 4.51
Average	1 : 4.35(14)

a: The molar ratio was calculated by the integral ratio of signal *d* to $CHCl_3$

Table S14. The number of $CHCl_3$ molecules encapsulated in a unit cell determined by ^{1}H NMR spectroscopy

$CHCl_3$ in a unit cell	$CHCl_3$-occupied void space (Å^3, %)
70	5250, 32

A unit cell is composed of 16 Pd_3-complexes. The volume of $CHCl_3$ was calculated on Spartan 06 (75 Å^3) and the equilibrium geometry was determined by DFT calculation. (B3LYP 6-31G*). The total volume of the void space of a unit cell is 16173 Å^3 calculated by PLATON.

Thermogravimetric analysis of MMF-3

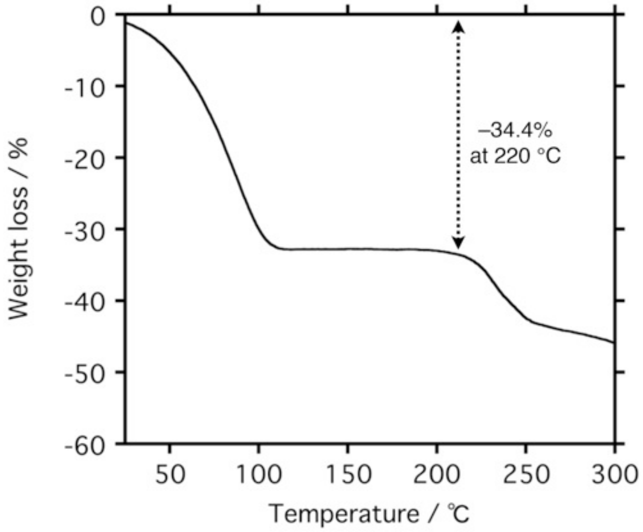

Figure S18. Thermogravimetric analysis of MMF-3. Measurement condition: 22 ~ 300 °C, 5 K/min, N_2 flow 150 mL/min. The 34% weight loss was approximately equal to the amount of solvents included in the crystals (31%).

Powder X-ray diffractions of rac-MMF-2 after soaked in $CHCl_3$

Conditions

Entry	Temp. (°C)	Time	Results
1	50	3 days	MMF-2 + MMF-3
2	50	7 days	MMF-3
3[a]	120	1 h	MMF-3
4[a]	120	2 h	MMF-3

a: Under microwave irradiation

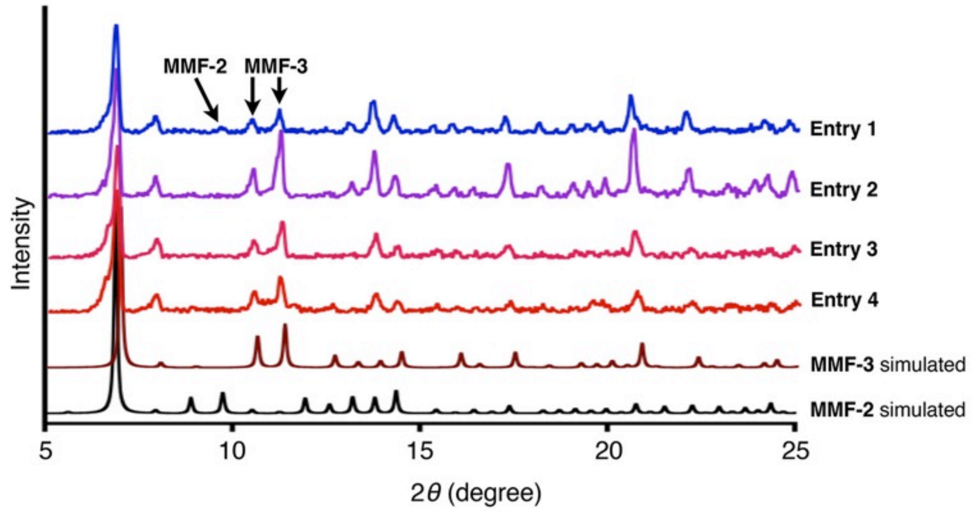

Figure S19. Powder X-ray diffractions of *rac*-MMF-2 after soaked in $CHCl_3$ under different conditions.

9. References for electronic supplementary information

1. Sheldrick, G. M. *SHELXL-97, Program for refinement of crystal structure* (University of Göttingen, Göttingen, Germany, 1997).
2. Spek, A. L. *PLATON, A Multipurpose Crystallographic Tool* (Utrecht University, Utrecht, The Netherlands, 2001).
3. Turner, M. J., McKinnon, J. J., Jayatilaka, D. & Spackman, M. A. Visualisation and characterisation of voids in crystalline materials. *CrystEngComm*. **13**, 1804–1813 (2011).
4. Perrin, C. L. & Dwyer, T. J. Application of two-dimensional NMR to kinetics of chemical exchange. *Chem. Rev.* **90**, 935–967 (1990).
5. Zuccaccia, D., Pirondini, L., Pinalli, R., Dalcanale, E. & Macchioni, A. Dynamic and structural NMR studies of cavitand-based coordination cages. *J. Am. Chem. Soc.* **127**, 7025–7032 (2005).
